# Perinatal outcomes associated with pre-exposure prophylaxis for HIV prevention during pregnancy: a systematic review and meta-analysis

**DOI:** 10.1016/j.eclinm.2024.102532

**Published:** 2024-03-19

**Authors:** Alison Erlwanger, Isabelle Rocroi, Shona Kirtley, Joris Hemelaar

**Affiliations:** a National Perinatal Epidemiology Unit, Infectious Disease Epidemiology Unit, Oxford Population Health, Nuffield Department of Population Health, University of Oxford, Oxford, UK; b Centre for Statistics in Medicine, Nuffield Department of Orthopaedics, Rheumatology and Musculoskeletal Sciences, University of Oxford, Oxford, UK

**Keywords:** HIV, Pre-exposure prophylaxis, Pregnancy, Preterm birth, Small for gestational age, Low birthweight, Neonatal death

## Abstract

**Background:**

The World Health Organization (WHO) recommends tenofovir disoproxil fumarate (TDF)-based oral pre-exposure prophylaxis (PrEP), the dapivirine vaginal ring, and long-acting intramuscular injectable cabotegravir (CAB-LA) for HIV prevention in populations at substantial risk of HIV infection. Pregnancy is a period of elevated risk of maternal HIV infection and transmission to the infant. This systematic review and meta-analysis assessed the risk of adverse perinatal outcomes among HIV-negative pregnant women with exposure to any PrEP modality.

**Methods:**

We conducted a systematic review by searching Medline, EMBASE, CINAHL, Global Health, the Cochrane Library, WHO ICTR, ISRCTN, PACTR, and ClinicalTrials.gov for studies published between 1 January 2000 and 29 August 2023. We included studies reporting on the association of antenatal exposure to any PrEP modality with 13 perinatal outcomes: preterm birth (PTB), very PTB, spontaneous PTB, spontaneous very PTB, low birthweight (LBW), very LBW, term LBW, preterm LBW, small for gestational age (SGA), very SGA, miscarriage, stillbirth, or neonatal death (NND). Quality assessments of included studies were performed. Fixed-effect meta-analyses were conducted to calculate odds ratios (ORs) and 95% confidence intervals (95% CIs). The protocol is registered with PROSPERO, CRD42022339825.

**Findings:**

Of 18,598 citations identified, 13 studies (eight randomised controlled trials (RCTs) and five cohort studies), assessing 8712 pregnant women in Africa, were included. Oral PrEP, compared to no PrEP, was not associated with PTB in meta-analyses of six RCTs (OR 0.73, 95% CI 0.43–1.26; *I*^*2*^ = 0.0%) or five unadjusted cohort studies (OR 0.84, 95% CI 0.69–1.03; *I*^*2*^ = 0.0%), but was associated with a reduced risk of PTB in three adjusted cohort studies (aOR 0.67; 95% CI 0.52–0.88, *I*^*2*^ = 0.0%). There was no association of oral PrEP with LBW, vLBW, SGA, or NND, compared to no PrEP. There was no association with PTB when oral TDF/emtricitabine (FTC) PrEP, oral TDF PrEP, and tenofovir vaginal gel were compared to each other. There was no association of the dapivirine vaginal ring with PTB or NND, compared to placebo or oral TDF/FTC PrEP. We found no data on CAB-LA.

**Interpretation:**

We found no evidence of adverse perinatal outcomes associated with PrEP exposure during pregnancy. Our findings support the WHO recommendation to provide oral PrEP to women of reproductive age and pregnant women. More data is needed to assess the safety of all PrEP modalities in pregnancy.

**Funding:**

None.

**Research in context**

**Evidence before this study**

Use of HIV pre-exposure prophylaxis (PrEP), including tenofovir disoproxil fumarate (TDF)-based oral PrEP, the dapivirine vaginal ring, and long-acting intramuscular injectable cabotegravir (CAB-LA), among HIV-negative pregnant women in populations with high HIV prevalence could play a crucial role in reducing maternal HIV incidence and vertical HIV transmission. We searched four literature databases (Medline, EMBASE, CINAHL, Global Health), the Cochrane Library, and four clinical trials databases (WHO ICTR, ISRCTN, PACTR, and ClinicalTrials.gov) for studies investigating the association of antenatal exposure to any PrEP modality with adverse perinatal outcomes, published between 1 January 2000 and 29 August 2023, using search terms for “HIV pre-exposure prophylaxis” and “perinatal outcome”. We found no meta-analysis of adverse perinatal outcomes associated with exposure to any PrEP modality during pregnancy, indicating a significant evidence gap.

**Added value of this study**

To fill this evidence gap, we conducted the first systematic review and meta-analysis to assess the association of antenatal exposure to any PrEP modality with thirteen specific adverse perinatal outcomes, including eight randomised controlled trials and five cohort studies, assessing 8712 pregnant women in Africa. We found that oral PrEP, compared to no PrEP, was not associated with preterm birth, low birthweight, very low birthweight, small for gestational age, or neonatal death. We found no association of the dapivirine vaginal ring with preterm birth or neonatal death, compared to placebo or oral PrEP, and we found no data on CAB-LA.

**Implications of all the available evidence**

We found no evidence of adverse perinatal outcomes associated with PrEP exposure during pregnancy, although there is low certainty of these findings because of the limited available data and low quality of the studies. This analysis supports the WHO recommendation that TDF-based oral PrEP is safe during pregnancy and more data is needed to assess the safety of the dapivirine ring and CAB-LA. With the expansion of PrEP programmes and with more PrEP modalities in development it is imperative to determine the safety profiles of different PrEP modalities to give women of reproductive age and pregnant women in high HIV prevalence settings adequate tools to safeguard their own health and the health of their babies.

## Introduction

Globally, 1.3 million people were newly infected with HIV in 2022.^1^ Women and adolescent girls in Africa had three times greater risk of HIV infection than their male counterparts.^1^ During pregnancy and the postpartum period, women have a more than two-fold increased risk of HIV infection in countries with a high HIV prevalence. Moreover, close to one third of vertical HIV transmissions in these settings are estimated to occur following maternal seroconversion during pregnancy.^2^

Untreated maternal HIV infection is associated with an increased risk of adverse perinatal outcomes, such as preterm birth (PTB), low birthweight (LBW), small for gestational age (SGA), and stillbirth.^3^ Antiretroviral therapy may also be associated with increased risk of adverse perinatal outcomes.^4^^,^^5^ Adverse perinatal outcomes are a major threat to meeting UN Sustainable Development Goal (SDG) target 3.2 to end preventable neonatal and child mortality, particularly in African countries with the highest mortality rates.^6^ Globally, PTB is the leading cause of death in children under 5 years old^7^ and 21.9% of neonatal deaths are attributable to infants born SGA.^8^ Approximately 80% of neonatal deaths annually are in children with LBW.^9^ Interventions to prevent maternal HIV infection during pregnancy are, therefore, critical to safeguard both maternal and child health.

The World Health Organization (WHO) recommends a daily tenofovir disoproxil fumarate (TDF)-based oral pill, the dapivirine vaginal ring (replaced monthly), and long-acting intramuscular injectable cabotegravir (CAB-LA) (received four to eight weeks apart) as part of combination HIV prevention strategies for populations at substantial risk of HIV infection.^10^^,^^11^ In a meta-analysis that included cisgender women, oral pre-exposure prophylaxis (PrEP) was shown to reduce the risk of HIV infection by 51% across studies, however, effectiveness varied when studies were stratified by adherence level from no effect (low adherence) to a reduction of 70% (high adherence).^12^ The dapivirine ring reduced the risk of HIV transmission in cisgender women by approximately 30% in a study with a relatively high adherence level (>82% blood plasma drug detection), while offering a more discrete alternative to oral PrEP.^13^ In efficacy studies, CAB-LA was associated with a 79% HIV risk reduction compared to TDF-based oral PrEP.^11^ Additional antiretroviral (ARV)-based HIV prevention methods, including implants and alternative oral formulations, at various stages of development and evaluation are not yet available for pregnant women, necessitating robust safety data for the modalities that are approved.^14^

Oral PrEP containing TDF has been found to be safe in studies that included cisgender heterosexual men and women, transgender women, men who have sex with men, serodiscordant couples, and people who inject drugs.^12^ These randomised controlled trials (RCTs) found that the rate of adverse events, including abnormal renal, liver and bone parameters, did not differ between oral TDF-based PrEP and placebo groups. Fewer studies have been conducted on other types of PrEP, but existing evidence shows a favourable safety profile for the dapivirine ring in cisgender women^15^ and CAB-LA in both men and women.^11^ Safety data on PrEP used in pregnancy are limited as study drugs are commonly stopped for participants who become pregnant in RCTs for novel PrEP modalities.

PrEP use among HIV-negative pregnant women in populations with high HIV prevalence can play a crucial role in reducing maternal HIV incidence and vertical HIV transmission. A modelling study estimated that offering PrEP to pregnant and breastfeeding women in Africa is a very cost-effective intervention that could avert 303 maternal HIV infections and 78 infant infections per 10,000 HIV-negative pregnant women on PrEP.^16^ However, concerns about the safety of PrEP in pregnancy have been a barrier to including pregnant women in PrEP studies and subsequently during the roll-out of PrEP among women of reproductive age and pregnant women.^14^^,^^17^ A previous systematic review (without meta-analysis), which included data up until 2019, reported no evidence of adverse perinatal outcomes associated with antenatal oral PrEP use, although limited data were available at the time and additional studies have been published since.^18^ To our knowledge, no meta-analysis has analysed perinatal outcomes associated with antenatal exposure to any PrEP modality.

The evidence gap regarding the safety of PrEP in pregnancy threatens integration of PrEP into routine care and scale-up to improve accessibility. For example, many countries allow nurses to prescribe combination anti-retroviral therapy (cART) to people living with HIV, yet few countries allow nurses to prescribe PrEP, despite the pharmacologic similarities between cART and oral PrEP.^19^ One reason for this discrepancy in national PrEP-prescribing policies is the dearth of evidence on the safety of PrEP in pregnancy.^20^ To help fill this evidence gap, we conducted a systematic review and meta-analysis to assess the association of antenatal exposure to any PrEP modality with specific adverse perinatal outcomes among HIV-negative pregnant women.

## Methods

### Search strategy and screening

The systematic review and meta-analysis protocol was developed in accordance with Cochrane guidelines^21^ and registered online (PROSPERO, CRD42022339825). Four literature databases (Medline, CINAHL, (EBSCOhost), Global Health (Ovid), EMBASE (Ovid)), the Cochrane Library, and four clinical trial databases (WHO ICTR, ISRCTN, PACTR, and ClinicalTrials.gov) were searched for entries published between 1 January 2000 and 29 August 2023. A comprehensive search strategy (Appendix 2) was developed and adapted for each literature database by a specialist librarian (SK) to include free text and controlled vocabulary search terms for “HIV pre-exposure prophylaxis”, “pregnancy outcome”, and “specific perinatal outcomes”. The Cochrane Library and trial databases were searched using broad filters for “HIV”, “HIV prevention”, and “infectious disease”. No restrictions were applied for methods, country, or language. Full-text articles, abstracts, conference poster presentations, and protocols reporting relevant data were considered.

Retrieved literature database citations were imported into Covidence systematic review software (Veritas Health Innovation, Melbourne, Australia) and deduplicated, while Cochrane review and protocol database citations were exported into Excel files. Title and abstract screening were conducted by two independent reviewers (AE and IR) to identify relevant articles for full-text review. Full-text documents were assessed against study eligibility criteria by two independent reviewers (AE and IR) to select articles for inclusion in the meta-analysis. References from all citations in the full-text review were screened to identify additional relevant studies. PubMed and Google were used to search for articles related to relevant protocols that did not reference manuscript publications or contain results.

### Study eligibility criteria

Studies that reported adverse perinatal outcomes associated with exposure to any PrEP modality during pregnancy were included based on the following eligibility criteria: study design (RCTs, cohort studies, case–control studies, or cross-sectional studies), population (HIV-negative pregnant women), exposure (any PrEP modality), and comparator (no PrEP or a different type of PrEP). All PrEP modalities (oral pill, gel, ring, injectable, patch, implant, vaginal insert, or film), drug formulations, timing of initiation (preconception, peri-conception, first, second, or third trimester), or durations of use were included. Studies were excluded if the PrEP modality, drug, or timing of exposure were undefined or if additional treatments were imbalanced between PrEP and comparator groups. Single arm studies, without a comparator group, were excluded.

Thirteen primary outcomes were assessed: preterm birth (PTB, birth <37^+0^ weeks' gestation); very PTB (vPTB, birth <32^+0^ weeks' gestation); spontaneous PTB (sPTB, spontaneous birth <37^+0^ weeks' gestation); spontaneous very PTB (svPTB, spontaneous birth <32^+0^ weeks' gestation); low birthweight (LBW, birthweight <2500 g); very LBW (vLBW, birthweight <1500 g); term LBW (LBW at ≥37^+0^ weeks' gestation); preterm LBW (LBW at <37^+0^ weeks' gestation); small for gestational age (SGA, birthweight for gestational age <10^th^ centile, based on any chart); very SGA (vSGA, birthweight for gestational age <3^rd^ centile, based on any chart); miscarriage (spontaneous expulsion of foetus at <24^+0^ weeks’ gestation); stillbirth (delivery of an infant without any signs of life with birthweight >1000 g or gestational age >24^+0^ weeks or body length >35 cm); neonatal death (NND, the death of an infant in the first 28 days of life).^3^ Maternal HIV infection was assessed as a secondary outcome. Studies were excluded if the outcome of interest was not defined or if the definition differed from the review protocol. If different outcomes from the same trial/cohort were reported in different publications, each was included. Inclusion and exclusion ambiguities and disagreements were resolved through discussion with the senior investigator (JH).

### Data extraction

Two reviewers (AE and IR) extracted data on the study characteristics, populations, PrEP exposure and comparator groups, and perinatal outcomes. Both dichotomous outcome frequencies and measures of association (odds ratios (ORs) and 95% confidence intervals (95% CIs)) were extracted. When information was not available in the primary publication, the reviewers searched for study protocols and related articles on ClinicalTrials.gov, research group websites, article references, and PubMed. Reviewers contacted 20 authors for additional information regarding relevant studies that did not have published outcome data or if other key information (e.g. outcome definitions) was missing.

### Quality and risk of bias assessments

Two reviewers (AE and IR) assessed the risk of bias in RCTs and the quality of cohort studies. RCTs were assessed using the Cochrane Risk of Bias 2 tool across five domains: randomisation process, deviations from the intended intervention, missing outcome data, measurement of the outcome, and selection of reported result. The overall risk of bias was classified as ‘low’, ‘some concerns’, or ‘high risk’ using predefined criteria (Appendix 3). The methodological quality of cohort studies was assessed with an adapted Newcastle–Ottawa tool across three categories: selection of the exposed and comparator cohorts, comparability of the cohorts, and outcome measurement and completeness of follow-up. According to predefined criteria, cohort studies were classified as ‘good’, ‘average’, or ‘poor’ quality (Appendix 4).

### Statistical analysis

A meta-analysis was conducted if two or more studies reported data for the same perinatal outcome for the same PrEP exposure comparison. Data from single studies that could not be meta-analysed are also reported. RCTs and cohort studies were analysed separately to reduce methodological heterogeneity. Fixed-effect models using inverse variance weighting were used to calculate summary effect estimates (ORs and 95% CIs) from dichotomous outcome frequencies for all RCTs and unadjusted cohort studies. Where available, adjusted odds ratios were converted to the log scale to be meta-analysed and then exponentiated for reporting. Random-effects meta-analysis results showed no material differences from the fixed-effect results (Appendix 8). Meta-analyses are represented in forest plots displaying individual studies, weighted summary effect estimates, and 95% CIs.

The *I*^*2*^ statistic was used to quantify heterogeneity due to clinical and methodological variability between studies. Heterogeneity was classified as none (<25%), low (25%–49%), moderate (50–74%) or high (>75%). Subgroup analyses were planned *a priori* to consider country income status and study quality assessment, but these analyses could not be performed because all studies were conducted in low- and middle-income African countries, there was too little diversity in study quality and/or too few studies. Funnel plots were used to assess small study effects (Appendix 7).

All analyses were conducted in Stata versions 17 and 18 (College Station, Texas, USA). Reporting is in accordance with the Preferred Reporting Items for Systematic Reviews and Meta-Analysis (PRISMA) guidelines (Appendix 1).^22^

### Role of funding source

This study received no funding.

## Results

### Systematic review

**Fig. 1:**
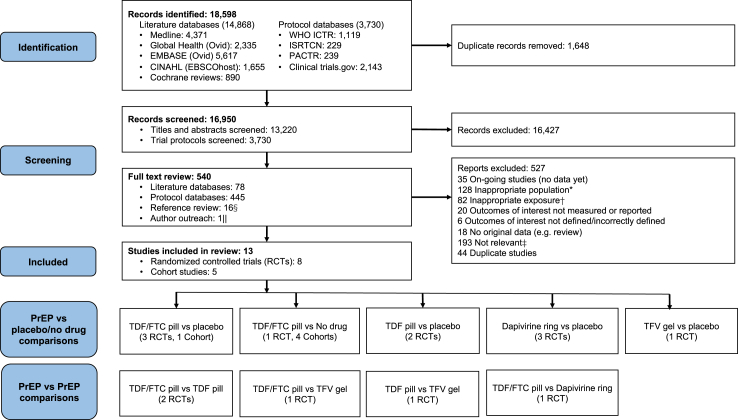
**Study selection**. §16 additional records were identified from reviewing the references of articles selected for a full text review. ||One study was identified after contact with an author about a different study. ∗For example, women were not pregnant. †For example, formulation demonstrated to be ineffective e.g. 0.5% PRO2000 gel. ‡For example, behavioural HIV prevention intervention. Abbreviations: CINAHL, Cumulated Index to Nursing and Allied Health Literature; EBSCO, Elton B. Stephens Company; EMBASE, Excerpta Medica database; FTC, emtricitabine; ISRCTN, International Standard Randomised Controlled Trial Number; PACTR, Pan African Clinical Trials Registry; RCT, randomised controlled trial; TDF, tenofovir disoproxil fumarate; TFV, tenofovir; WHO ICTR, World Health Organization International Clinical Trials Registry Platform.

The PRISMA flowchart summarizes the systematic review process (Fig. 1). The literature and protocol searches identified 18,598 citations, 523 of which were selected for a full text and reference review. An additional 16 relevant citations were identified from reviewing the references of articles and protocols. Through author outreach one additional study was identified^23^ and further unpublished data was received for five studies.^23^, ^24^, ^25^, ^26^, ^27^ Thirteen studies met the meta-analysis eligibility criteria.^23^, ^24^, ^25^, ^26^, ^27^, ^28^, ^29^, ^30^, ^31^, ^32^, ^33^, ^34^, ^35^ Thirty-five potentially relevant studies were excluded because they are ongoing and did not have any data available at present.

### Study characteristics

Characteristics of included studies are summarised in Table 1. The thirteen included studies had data for 8712 pregnant women from six east and southern African countries across eight RCTs, four prospective cohort studies, and one retrospective cohort study. Recruitment in six studies exclusively targeted pregnant women or women trying to conceive.^24^^,^^27^^,^^30^^,^^32^^,^^33^^,^^35^ Women in the remaining seven studies fell pregnant while participating in other PrEP studies.^23^^,^^25^, ^26^, ^27^^,^^29^^,^^31^^,^^34^ All RCTs had a high risk of bias, mainly due to a risk of outcome measurement bias (suboptimal method to assess gestational age, which increases the risk of misclassification bias), while three cohort studies were average quality and two were poor quality (Table 1, Appendix 3 and 4). Four cohort studies conducted a risk factor analysis to identify characteristics that were statistically significantly different between the PrEP and comparator groups, with three of the cohort studies subsequently conducting a regression analysis that accounted for confounding by reporting adjusted ORs (details on risk factor analysis and adjustment variables in Appendix 4.4).

**Table 1:** Study characteristics.

Study^a^	Trial/cohort	Country	Study design	Number of pregnant women analysed	Recruiting period	Participant characteristics^b^	Study quality
Randomised controlled trials (RCTs)							
Bunge 2015^27^^c^	VOICE (MTN-003)	South Africa (76%), Zimbabwe (13%), Uganda (12%)	5-arm phase IIb	428	2009–2011	Women who fell pregnant during the VOICE trial (5029 women in parent study). Women in the pregnancy subgroup: median age of 23 years (IQR 21–27), and 91.8% had secondary education or higher. 57% delivered in a hospital and 23% in a health centre.	High risk of bias
Bunge 2023^28^	DELIVER (MTN-042)	Malawi, South Africa, Uganda, Zimbabwe	2-arm phase IIIb	307	2/2020–3/2022	Pregnant women were recruited in the third trimester of pregnancy from four study sites in urban and peri-urban areas. Three cohorts were recruited at different gestational ages (Cohort 1 at 36–38 weeks, Cohort 2 at 30–35 weeks, and Cohort 3 at 12–29 weeks). We include data from the first two study cohorts, for which data was available. Cohort 1 had a median age of 25 years (IQR 21–28).	High risk of bias
Callahan 2015^26^^c^	FEM-PrEP	South Africa, Tanzania, Kenya	2-arm	115	6/2009–4/2011	Women who fell pregnant during the FEM-PrEP study (2120 women in parent study recruited at four sites). Women in the parent study: median age 23, 69.2% not married, average of 10.4 years of education, 72.3% had a previous pregnancy, and 12.6% reported engaging in transactional sex.	High risk of bias
Kusemererwa 2018^25^^c^	The Ring Study (IPM 027)	Uganda	2-arm phase III	25	2013–2016	Women who fell pregnant during The Ring Study (197 women in parent study were recruited from HIV hot spots (e.g. bars and hair salons) in small townships in south western Uganda). Women in the parent study had a mean age of 28 years (SD 6.6).	High risk of bias
Makanani 2018^13^^,^^*29*^	MTN-020/MTN 016-ASPIRE	South Africa (53%), Zimbabwe (27%), Uganda (15%), Malawi (5%)	2-arm phase III	169	2012–2014	Women who fell pregnant during MTN 020/MTN 016 study (2629 women in parent study recruited at 15 sites). Women in the pregnancy subgroup: median age of 23 years (IQR 21–27), 38% married, and 56% had secondary education or higher. 29% and 13% of the intervention and comparator group respectively had previously had an STI infection. Twins included.	High risk of bias
Moodley 2023^*30*^^,^^36^	CAP016	South Africa	2-arm	540	9/2017–12/2019^d^	Pregnant women (all black South African) were recruited at one site. Women had a median age of 23 years (IQR 20–26), BMI at enrolment of 27.9 (IQR 24.3–31.9) in the intervention group versus 26.6 (23.2–31.3) in the comparator group. 47% (intervention) and 46% (comparator) had a gravidity of one. Twins excluded.	High risk of bias
Mugo 2014^31^	Partners PrEP study	Kenya, Uganda	3-arm phase III	288	7/2008–11/2010	Women who fell pregnant during the Partners PrEP study (4747 HIV-negative women in serodiscordant couples in the parent study were recruited at nine sites). Women in the pregnancy subgroup: median age of 33 years (IQR 28–28), education duration of 6 years (IQR 3–8). 95.5% had a previous child. Twins included.	High risk of bias
Nel 2016^23^^c^	The Ring Study (IPM 027)	South Africa, Uganda	2-arm, phase III	68	2012–2016	Women who fell pregnant during The Ring Study (1959 women in parent study were recruited at seven sites). Women in the parent study had a mean age of 26 years (range 18–45), 89.2% were single and 98.2% reported having a single main sex partner.	High risk of bias
Cohort studies							
Davey 2022^*32*^^,^^37^	PrEP-PP	South Africa	Prospective (on-going)	807	8/2019–10/2021	Pregnant HIV-negative adolescent girls and women were recruited at one site in an urban area. Women had a median age of 26 years (IQR 22–31). 1% had a known HIV-positive partner and 30% did not know their partner's status. In the past year, 31% reported an STI infection, 50% report alcohol and/or drug use, and 12% experienced IPV. Twins included.	Poor quality
Dettinger 2019^*33*^^,^^38^^,^^39^	PrIYA	Kenya	Retrospective	1530	6/2017–6/2018	Mother and infant pairs receiving postnatal care were recruited at 16 sites. Women had a median age of 24 years (IQR 21–28). 1% reported a previous syphilis infection while 5.8% and 1.1% of women in the intervention and comparator group respectively had experienced IPV (p < 0.05).	Average quality
Dettinger 2020^35^	PrIMA	Kenya	Prospective^e^ (on-going)	4261	1/2018–4/2019	Women who completed the PrIMA study (recruiting pregnant women at 20 sites in high HIV prevalence (i.e. >20%) areas) were invited to participate in an open label study. In the intervention arm, women had a median age of 25.1 years (IQR 21.0–30.0), 5.2% had previously had an STI, 18.8% knew their partner to be HIV positive, 3.6% engaged in transactional sex, and 9% experienced IPV. In the comparator arm, women had a median age of 23.9 years (IQR 20.9–28.1), 1.9% had previously had an STI, 1.3% knew their partner to be HIV positive, 1.4% engaged in transactional sex, and 4.9% experienced IPV (p < 0.001 for all comparisons).	Average quality
Heffron 2018^34^	Control–Partners PrEP study^f^ Exposed–Partners Demonstration Project	Kenya, Uganda	Prospective	118	Control–7/2008–11/2010^f^ Exposed–11/2012–8/2014	Women in the control arm (nine recruitment sites) had fallen pregnant during the Partners PrEP phase III RCT of serodiscordant couples. They had a median age of 28 years. Women in the exposed arm (four recruitment sites) had fallen pregnant during an open-label PrEP demonstration project for high-risk HIV serodiscordant couples. They had a median age of 25 years and a higher HIV risk score than the comparator group. Twins included in both arms.	Average quality
Matthews 2018^24^^c^	SYMBOL study	Uganda	Prospective	56	11/2016 - end not specified	Women were part of the SYMBOL study of serodiscordant couples trying to conceive who were recruited at one site. 49% of participants in the parent study were men and 91% of participants reported having HIV-negative pregnancy partners.	Poor quality

Characteristics of studies included in the meta-analysis are summarised.

Abbreviations: BMI, body mass index; IPV, intimate partner violence; IQR, interquartile range; SD, standard deviation; STI, sexually transmitted infection.

a Where multiple references are cited, the italicized citations were identified through the systematic literature review. Additional sources of information are also cited.^13^^,^^36^, ^37^, ^38^, ^39^ Two included studies were from a single RCT (IPM 027), but reported different outcomes.^1^^,^^2^ Two cohort studies were ongoing, but data was available for over 90% of those recruited.^32^^,^^35^

b Where available, information was extracted for: number of recruitment sites, area (urban versus rural), catchment area HIV prevalence, maternal age, prior STI infections, smoking status, alcohol use, intravenous drug use, behavioural and social HIV risk factors, race, BMI/weight, and multiple pregnancies. Characteristics are reported for the pregnant women analysed, which are sometimes a subgroup of a larger study. If data on the pregnant subgroups were not available, the characteristics for the parent trial or cohort are reported.

c Some or all of study data was unpublished and provided directly by the author.

d Premature suspension of enrolment when South Africa's PrEP guidelines were expanded to include pregnant women.

e Dettinger 2020 study is nested in a cluster randomised RCT.

f The control and exposed arms of the Heffron 2018 study were taken from two different studies conducted at different times.

Details on the PrEP modalities, initiation, retention, and adherence, the comparator group(s), and perinatal outcomes are summarised in Table 2. Ten studies (five RCTs and all five cohort studies) had at least one oral PrEP arm, four studies (all RCTs) had a dapivirine vaginal ring arm, and one study (RCT) had a tenofovir (TFV) vaginal gel arm. No studies reported on long-acting injectable PrEP, including CAB-LA, or other PrEP modalities. Twelve studies had a comparator arm that was not exposed to any type of PrEP (seven placebo and five no drug comparator arms). Three studies had more than one PrEP exposure arm. In six studies (all RCTs) women initiated PrEP preconception and PrEP was discontinued once pregnancy was detected during routine monthly follow-up visits. There was no data on fetal mortality related to any PrEP modality.

**Table 2:** HIV pre-exposure prophylaxis (PrEP) modalities, comparators, and adverse perinatal outcomes.

Study^a^	PrEP (modality, dose, frequency)	PrEP initiation and retention	PrEP adherence	Comparator	Outcomes
Randomised Controlled Trials					
Bunge 2015^27^^c^	Daily oral TDF pill (arm 1) Daily oral TDF/FTC pill (arm 2) Daily 1% TFV vaginal gel (arm 3)	Initiated preconception and discontinued when pregnancy was detected as part of monthly study monitoring tests.	Low adherence: 9–16% of pregnant women in study took the study drug in the week prior to drug sampling.	Placebo	PTB
Bunge 2023^28^	Daily oral TDF/FTC (300 mg/200 mg)	Initiated late in pregnancy (cohort 1: 36–38 weeks; cohort 2: 30–35 weeks) and continued until delivery or 41^+6^ weeks gestation.	Most participants had evidence of product exposure during their time in the study. >95% of expected maternal visits completed and pregnancy outcomes available for >98% of participants (adherence analyses ongoing).	Dapivirine vaginal ring	PTB, NND, maternal HIV
Callahan 2015^26^^c^	Daily oral TDF/FTC (300 mg/200 mg)	Initiated preconception among women assessed to be at high HIV risk. PrEP discontinued when pregnancy was detected as part of monthly study monitoring tests.	Low adherence (using plasma samples from 4-week intervals).	Placebo	PTB
Kusemererwa 2018^25^^c^	Dapivirine vaginal ring (replaced monthly)	Initiated preconception among women assessed to be at high HIV risk. PrEP discontinued when pregnancy was detected as part of monthly study monitoring tests.	No information reported.	Placebo	NND
Makanani 2018^13^^,^^*29*^	Dapivirine vaginal ring (replaced monthly)	Initiated preconception and discontinued when pregnancy was detected as part of monthly study monitoring tests.	Drug was detected at prespecified acceptable levels in 82% of samples in the parent study arm (i.e. including non-pregnant women)^b^.	Placebo	PTB
Moodley 2023^*30*^^,^^36^	Daily oral TDF/FTC (300 mg/200 mg)	Initiated in second trimester (median GA 19 weeks); PrEP discontinued after breastfeeding cessation in exposed arm.	Mean calculated adherence of 87% (SD 24) at 4 weeks, 93% (SD 17) at 8 weeks, 94% (SD 16) at 12 weeks, and 95% (SD 17) at 16 weeks after initiation (calculated using pill count).	No drug	PTB, LBW, vLBW, SGA, NND, maternal HIV
Mugo 2014^31^	Daily oral TDF/FTC (arm 1) (300 mg/200 mg) Daily oral TDF (arm 2) (300 mg)	Initiated preconception and discontinued when pregnancy was detected as part of monthly study monitoring tests.	Estimated PrEP exposure of approximately 6 weeks or less from conception to pregnancy detection.	Placebo	PTB
Nel 2016^23^^c^	Dapivirine vaginal ring (replaced monthly) (25 mg)	Initiated preconception and discontinued when pregnancy was detected as part of monthly study monitoring tests.	83% of the returned rings had a concentration of 23.5 mg or less of dapivirine. Dapivirine was detected at prespecified level or above in 84% of samples taken every 4 weeks.	Placebo	PTB
Cohort studies					
Davey 2022^*32*^^,^^37^	Daily oral TDF/FTC (300 mg/200 mg)	Offered as part of routine care at first ANC visit when median gestational age was 21 weeks (IQR 13–31); 84% initiated same day; PrEP stopped when patients became lost to follow up.	66% and 58% returned at 1 month and 3 months for prescription, respectively; 46% taking PrEP at 3 months reported missing one or more daily doses.	No drug	PTB, LBW, SGA, NND
Dettinger 2019^*33*^^,^^38^^,^^39^	Daily oral TDF/FTC (300 mg/200 mg)	Initiated in maternal and child health clinics and family planning clinics. 6% initiated in first trimester, 57% in second trimester, 38% in third trimester. Patients decided when to stop.	58% had more than 45 days between initiation and discontinuation or date of birth.	No drug	PTB, LBW
Dettinger 2020^35^	Daily oral TDF/FTC (300 mg/200 mg)	Initiated during pregnancy with median gestational age of 24 weeks (IQR 20–28); 10 sites offered PrEP to all women and 10 sites targeted offer based on risk level. Patients decided when to stop.	No information reported.	No drug	PTB, LBW, SGA
Heffron 2018^34^	Daily oral TDF/FTC (300 mg/200 mg)	Initiated preconception and patients decided when to stop.	73% were dispensed PrEP at least once after confirming pregnancy; 52% took at least 80% of expected doses^d^; 74% of plasma samples detected TDF consistent with daily use.^d^	Placebo	PTB, maternal HIV
Matthews 2018^24^^c^	Daily oral TDF/FTC	Initiated preconception for patients in serodiscordant relationships trying to conceive.	85% took at least 80% of doses^d^ 20 of the 24 live births in the PrEP-initiating group were still taking PrEP at the time of delivery.	No drug	PTB, LBW, Preterm LBW

Details of PrEP exposure, comparators, and outcomes reported by included studies are summarised.

Abbreviations: ANC, antenatal clinic; GA, gestational age; FTC, emtricitabine; IQR, interquartile range; LBW, low birthweight (<2500 g), NND, neonatal death (death in first 28 days of life); PTB, preterm birth (<37^+0^ weeks' gestation); SD, standard deviation; SGA, small for gestational age (birthweight for gestational age <10^th^ centile); TDF, tenofovir disoproxil fumarate; TFV, tenofovir; vLBW, very low birthweight (<1500 g).

a Where multiple references are cited, the italicized citations were identified through the systematic literature review. Additional sources of information are also cited.^13^^,^^36^, ^37^, ^38^, ^39^ Two included studies were from a single RCT (IPM 027), but reported different outcomes.^1^^,^^2^ Two cohort studies were ongoing, but data was available for over 90% of those recruited.^32^^,^^35^

b Measured by plasma liquid chromatographic-tandem mass spectrometry.

c Some or all of study data was unpublished and provided directly by the author.

d Measured by electronic medication monitoring bottle caps.

### Oral PrEP compared to no oral PrEP

**Fig. 2:**
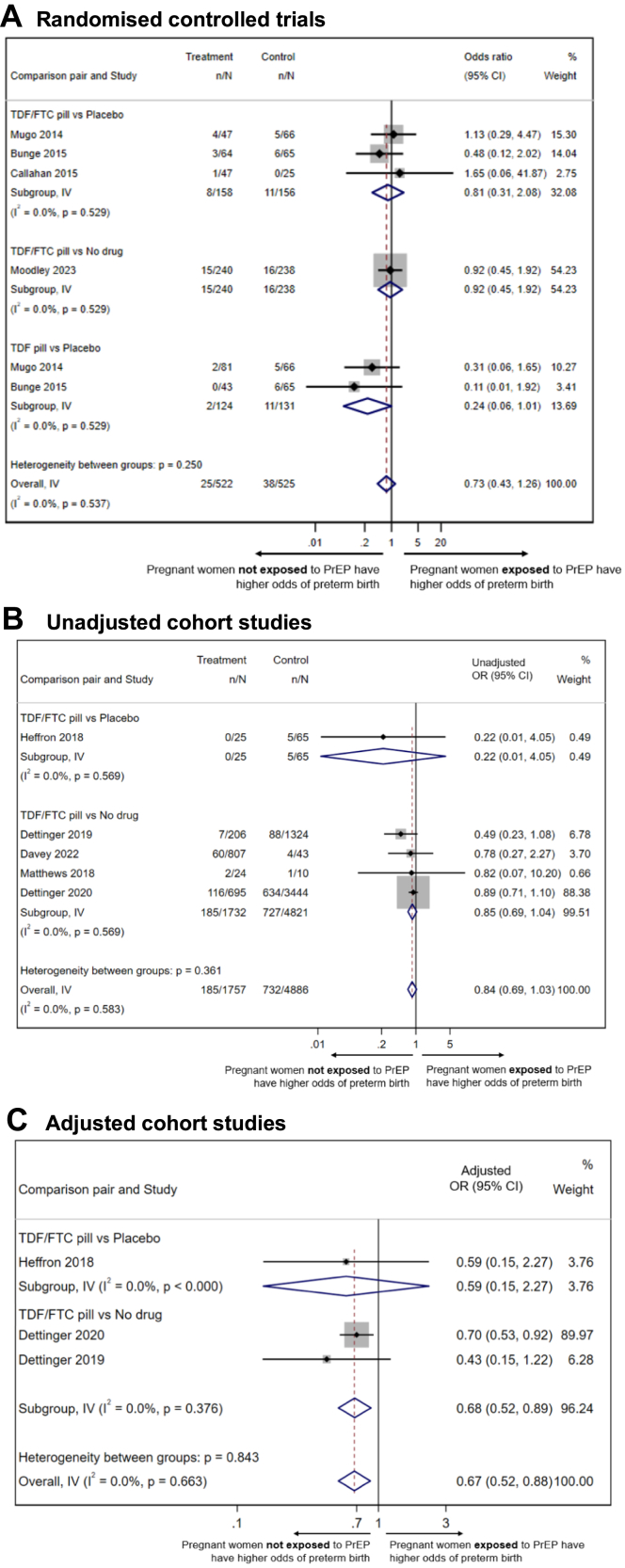
**Meta-analyses of association between oral PrEP exposure and preterm birth, compared to placebo or no drug**. **A. Randomised controlled trials. B. Unadjusted cohort studies. C. Adjusted cohort studies**. Meta-analyses of the association of antenatal oral PrEP exposure with preterm birth, compared to placebo or no drug. Fixed-effect meta-analysis models, inverse variance method. Grey shaded boxes display the relative contribution (% weight) of each individual study to the meta-analysis. Subgroup analysis by oral PrEP comparison pairs are shown, depending on available data: TDF/FTC pill versus placebo, TDF/FTC pill versus no drug, and TDF pill versus placebo. Odds Ratio (OR), 95% confidence intervals (95% CI), number of preterm birth (<37^+0^ weeks' gestation) events and total live births by arm (treatment and control), and weighting % are displayed. Abbreviations: FTC, emtricitabine; TDF, tenofovir disoproxil fumarate.

Fig. 2 displays meta-analyses assessing the association of oral PrEP with PTB, compared to no oral PrEP (either placebo or no drug). In the meta-analysis of six RCTs, including 1047 women, there was no association between oral PrEP and PTB, compared to no oral PrEP (OR 0.73, 95% CI 0.43–1.26; *I*^*2*^ = 0.0%) (Fig. 2A). In the meta-analysis of five unadjusted cohort studies (6643 women), there was no association between oral PrEP and PTB, compared to no oral PrEP (OR 0.84, 95% CI 0.69–1.03; *I*^*2*^ = 0.0%) (Fig. 2B). The meta-analysis of three of the cohort studies (5759 women) which adjusted for confounding (details on risk factor analysis and adjusted variables in Appendix 4.4) showed a statistically significant lower odds of PTB associated with oral PrEP, compared to no oral PrEP (aOR 0.67, 95% CI 0.52–0.88; *I*^*2*^ = 0.0%) (Fig. 2C).

**Fig. 3:**
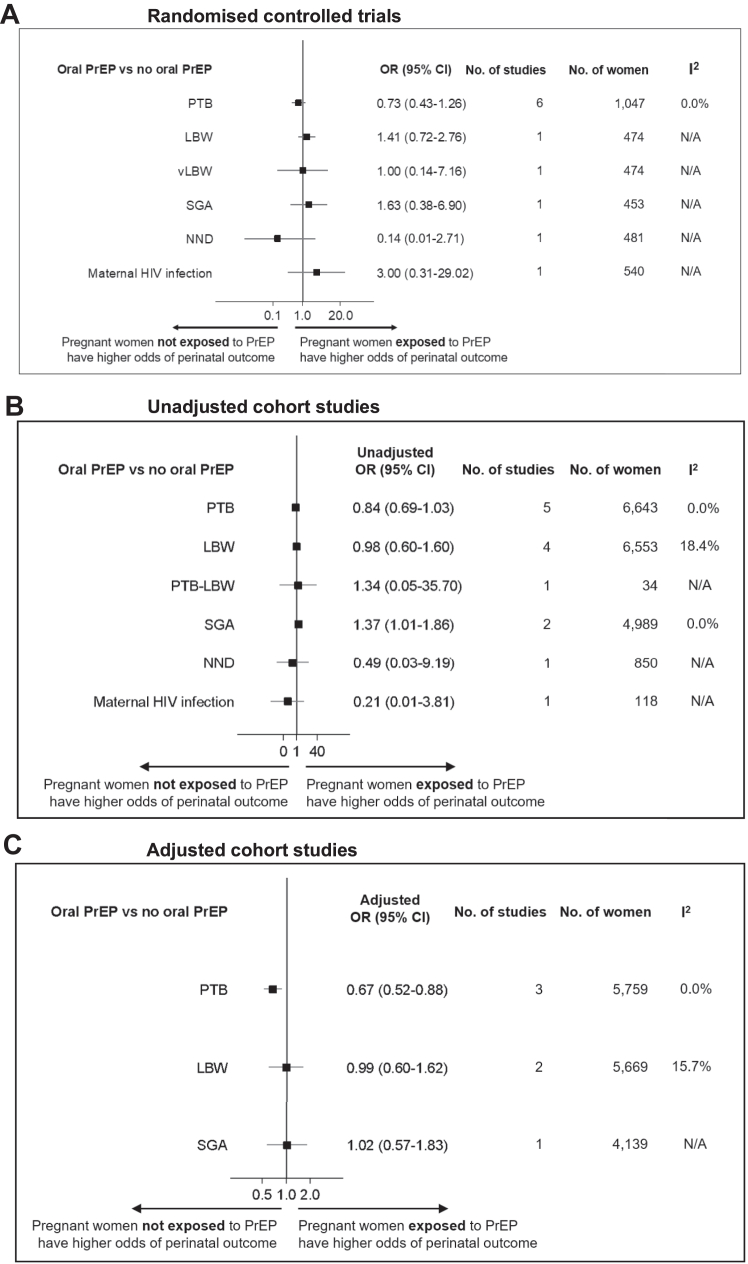
**Perinatal and maternal outcomes associated with oral PrEP exposure during pregnancy, compared to no oral PrEP. A. Randomised controlled trials. B. Unadjusted cohort studies. C. Adjusted cohort studies**. Association of antenatal oral PrEP exposure with perinatal and maternal outcomes, compared to no oral PrEP. Odds Ratio (OR), 95% confidence intervals (95% CI), number of studies included, number of women analysed, and *I*^*2*^ are displayed. If two or more studies reported data, a meta-analysis was performed (fixed-effect model, inverse variance method). See Appendices 5 and 6 for details of each analysis. Abbreviations: LBW, low birthweight (<2500 g); NND, neonatal death (death in first 28 days of life); PrEP, pre-exposure prophylaxis; PTB, preterm birth (<37^+0^ weeks' gestation); PTB-LBW, preterm low birthweight; SGA, small for gestational age (birthweight for gestational age <10^th^ centile); vLBW, very low birthweight (<1500 g).

Results from studies assessing the association between oral PrEP and multiple adverse perinatal outcomes, compared to no oral PrEP, are summarised in Fig. 3. If two or more studies reported data on the same perinatal outcome, a meta-analysis was performed. If only one study assessed an outcome, the result of that study is reported. One RCT found no association between oral PrEP and LBW (OR 1.41, 95% CI 0.72–2.76) or vLBW (OR 1.00, 95% CI 0.14–7.16), compared to no oral PrEP (Fig. 3A). In meta-analyses of four unadjusted cohort studies (6553 women) and two adjusted cohort studies (5669 women), there was no association between oral PrEP and LBW, compared to no oral PrEP (OR 0.98, 95% CI 0.60–1.60; *I*^*2*^ = 18.4% and aOR 0.99, 95% CI 0.60–1.62; *I*^*2*^ = 15.7%) (Fig. 3B and C). In one unadjusted cohort study, there was no association between oral PrEP and preterm LBW, compared to no oral PrEP (OR 1.34, 95% CI 0.05–35.70) (Fig. 3B).

There was no association of oral PrEP with SGA, compared to no oral PrEP, in one RCT (OR 1.63, 95% CI 0.38–6.90) (Fig. 3A). In a meta-analysis of two unadjusted cohort studies (4989 women), there was a statistically significant higher odds of SGA associated with oral PrEP, compared to no oral PrEP (OR 1.37, 95% CI 1.01–1.86; *I*^*2*^ = 0.0%) (Fig. 3B). However, when the largest cohort study (4139 women) was adjusted for confounding, there was no association between oral PrEP and SGA, compared to no oral PrEP (aOR 1.02, 95% CI 0.57–1.83) (Fig. 3C).

One RCT (OR 0.14, 95% CI 0.01–2.71) and one unadjusted cohort study (OR 0.49, 95% CI 0.03–9.19) found no association between oral PrEP and NND, compared to no oral PrEP (Fig. 3A and B). There was no association between oral PrEP and maternal HIV infection, compared to no oral PrEP, in one RCT (OR 3.00, 95% CI 0.31–29.02) (Fig. 3A) or one unadjusted cohort study (OR 0.21, 95% CI 0.01–3.81) (Fig. 3B).

### Dapivirine ring compared to placebo

**Fig. 4:**
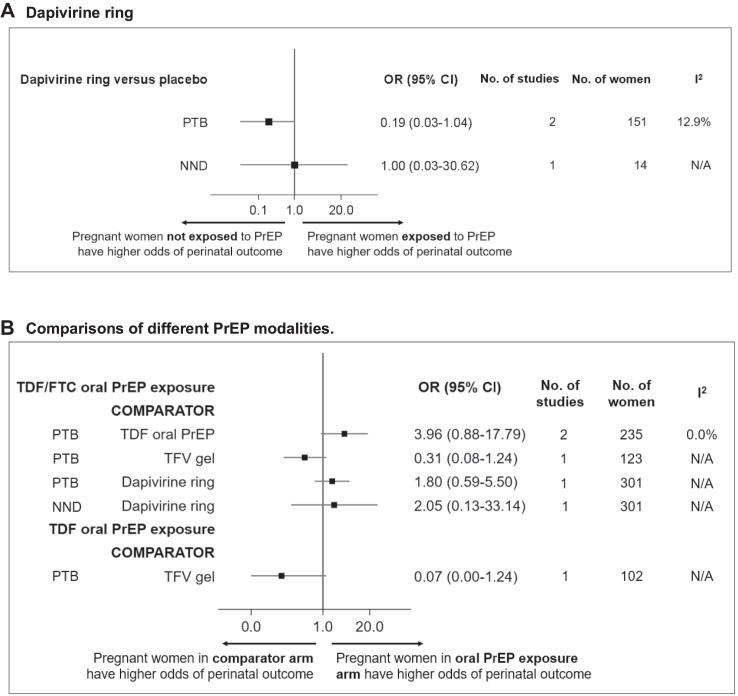
**Perinatal outcomes associated with different PrEP modalities. A. Dapivirine ring. B. Comparisons of different PrEP modalities**. Perinatal outcomes associated with different PrEP modalities in pregnancy. Data from randomised controlled trials. Odds Ratio (OR), 95% confidence intervals (95% CI), number of studies included, number of women analysed, and *I*^*2*^ are displayed. If two or more studies reported data, a meta-analysis was performed (fixed-effect model, inverse variance method). See Appendices 5 and 6 for details of each analysis. Abbreviations: NND, neonatal death (death in first 28 days of life); PrEP, pre-exposure prophylaxis; PTB, preterm birth (<37^+0^ weeks' gestation), TDF, tenofovir disoproxil fumarate; TFV, tenofovir.

Fig. 4A shows results from studies (all RCTs) assessing the association between dapivirine ring exposure and perinatal outcomes, compared to placebo. In a meta-analysis of two RCTs, there was no association of the dapivirine ring with PTB, compared to placebo (OR 0.19, 95% CI 0.03–1.04; *I*^*2*^ = 12.9%) (Fig. 4A). One RCT found no association between the dapivirine ring exposure and NND, compared to placebo (OR 1.00, 95% CI 0.03–30.62) (Fig. 4A).

### TFV gel compared to placebo

There was no association between 1% TFV vaginal gel and PTB, compared to placebo, in one RCT (OR 1.54, 95% CI 0.47–5.02) (Appendix 6).

### Comparisons of different PrEP modalities

Results from studies (all RCTs) comparing perinatal outcomes between different PrEP modalities are summarised in Fig. 4B. There was no association between oral TDF/emtricitabine (FTC) PrEP and PTB, compared to TDF oral PrEP, in a meta-analysis of two RCTs (OR 3.96, 95% CI 0.88–17.79; *I*^*2*^ = 0.0%) (Fig. 4B). There was also no association between TDF/FTC oral PrEP and PTB, compared to 1% TFV vaginal gel (OR 0.31, 95% CI 0.08–1.24) or compared to the dapivirine ring (OR 1.80, 95% CI 0.59–5.50) in individual RCTs (Fig. 4B). There was no association between TDF/FTC oral PrEP and NND, compared to the dapivirine ring (OR 2.05, 95% CI 0.13–33.14), or TDF oral PrEP and PTB, compared to 1% TFV vaginal gel (OR 0.07, 95% CI 0.00–1.24) in individual RCTs (Fig. 4B).

## Discussion

This is the first systematic review and meta-analysis of the association of antenatal exposure to any PrEP modality with adverse perinatal outcomes among HIV-negative pregnant women. We found that oral PrEP, compared to no PrEP, was not associated with PTB in meta-analyses of six RCTs and five unadjusted cohort studies, but was associated with a reduced risk of PTB in three cohort studies adjusted for confounding. There was no association of oral PrEP with LBW, vLBW, SGA, or NND, compared to no PrEP. There was no association with PTB when oral TDF/FTC PrEP, oral TDF PrEP, and TFV vaginal gel were compared to each other. There was no association of the dapivirine vaginal ring with PTB or NND, compared to placebo or oral TDF/FTC PrEP. We found no data on CAB-LA.

A previous systematic review, without meta-analysis, included two cohort studies which each showed a lower risk of PTB with antenatal exposure to oral PrEP, compared to no drug/placebo, although neither finding was statistically significant.^18^^,^^33^^,^^34^ Our meta-analysis included three additional cohort studies,^24^^,^^32^^,^^35^ which increased the number of women analysed from 1648 to 5124, and showed no association between oral PrEP and PTB. Excluding 863 women from two studies that did not report adjusted odds ratios,^24^^,^^32^ the adjusted meta-analysis showed a statistically significant lower odds of PTB associated with oral PrEP, compared to no PrEP. The three cohort studies were adjusted for factors that were found to be statistically significantly different between the PrEP and comparator groups, such as gestational age at PrEP screening, partner HIV status, maternal age, history of pregnancy loss and/or syphilis status. Our analogous meta-analysis of RCTs showed a similar protective effect of PrEP with a smaller sample size (1047 women), although it was not statistically significant.

Past systematic reviews and meta-analyses that focused on pregnant women living with HIV and/or Hepatitis B (HBV) on TDF-based treatment are inconsistent regarding the risk of PTB compared to non-TDF based regimens.^40^^,^^41^ One meta-analysis, including data from 7924 women from four studies of different study designs (two cohort studies, one cross-sectional study, and one RCT), found that TDF-based treatment lowered the risk of PTB compared to non-TDF based-regimens (RR 0.90, 95% CI 0.81–0.99; *I*^*2*^ = 59.1%).^40^ In contrast, another analysis reported that antenatal TDF/FTC treatment was not associated with PTB in one RCT (RR = 0.94, 95% CI 0.69–1.28).^41^ Both studies on antenatal TDF-based HIV or HBV treatment showed evidence of an increased risk of neonatal death, but were inconsistent regarding stillbirth (decreased risk^40^ versus no association^41^). One of the reviews found no difference between TDF-based and non-TDF-based regimens in risk of miscarriage, SGA, LBW, or very LBW.^40^ Meta-analysis results on antenatal TDF-based treatment for HIV or HBV must be interpreted with caution when considering their relevance to TDF-based PrEP because both HIV and HBV have independent impacts on perinatal outcomes.^3^^,^^42^ The safety threshold is also higher when weighting the costs and benefits of ARVs as a preventative measure in a relatively healthy population. However, these meta-analyses do highlight challenges with generating good quality safety data for ARV use in pregnancy, which also apply to PrEP. There are few good quality and adequately powered RCTs to assess perinatal outcomes. In six out of the eight RCTs included in our meta-analysis PrEP was initiated preconception and discontinued once women fell pregnant, in line with a broader trend to disproportionately exclude pregnant women from ARV trials.^43^

Our systematic review and meta-analysis has several strengths. This is the first meta-analysis of adverse perinatal outcomes associated with exposure to any PrEP modality in pregnancy. The protocol, with study inclusion and exclusion criteria, was published in advance to limit selection bias, and outcomes were clearly pre-defined to minimise misclassification bias. To give a complete overview of all available evidence, all relevant study designs, a broad range of perinatal outcomes, and all PrEP modalities (including those not (yet) WHO approved) were eligible, regardless of duration of PrEP exposure. The references of each relevant article were searched to find additional studies. Authors were contacted if studies had the potential to track pregnant women on PrEP, even if that was not the primary study objective, which led to the inclusion of additional studies and outcome data. This led to the most comprehensive data set on perinatal outcomes associated with antenatal exposure to any PrEP modality to date.

This study has some limitations. There was no data on CAB-LA in pregnancy or data on fetal mortality related to any PrEP modality. There were too few studies to conduct subgroup analyses by study quality or confidently interpret funnel plots for publication bias. Despite broad inclusion criteria (including all countries), there was no geographical or country income level diversity, as all eligible studies recruited from just six countries in eastern and southern Africa, which limits the external validity of our findings in other contexts. Timing of PrEP initiation, duration of use, and adherence level varied widely between studies, but there was insufficient data to explore how this might impact adverse perinatal outcomes. Only six of the included studies explicitly recruited pregnant women or women trying to conceive. In six RCTs women initiated PrEP preconception and PrEP was discontinued once pregnancy was detected during routine monthly follow-up visits, usually in the first trimester. Although the first trimester is crucial for organogenesis, the duration of PrEP exposure is likely to have been relatively short in these RCTs. In most of the other studies PrEP was initiated in the second or third trimester, with varying levels of adherence. Low adherence and exposure may bias findings towards showing no statistically significant difference in perinatal outcomes between PrEP and comparator groups, because their actual exposure levels would be relatively similar. Adherence may be even more important during pregnancy because, controlling for the level of adherence, the plasma concentration of TDF has been found to be lower in pregnant women compared to non-pregnant women on oral PrEP.^44^ The method to measure gestational age varied in each study and was not the gold standard (ultrasound <14 weeks gestation), which compromised the accuracy of PTB and SGA measurements, leading to an increased risk of misclassification bias impacting the overall quality of the studies, resulting in most studies having high risk of bias (RCTs) or poor quality (cohort studies). A final limitation is that cohort studies are subject to confounding. PrEP initiation criteria are based on being at higher risk of HIV infection,^10^ which means PrEP-exposed and unexposed populations are substantially different. In our meta-analysis the PrEP-exposed groups tended to have a higher prevalence of factors with an independent impact on perinatal outcomes, such as intimate partner violence^45^ and infection with syphilis.^46^ On the other hand, PrEP use in cohort studies may decrease the risk of adverse perinatal outcomes, because women visit clinics for refills and have more interactions with healthcare service providers, which may decrease the likelihood of adverse perinatal outcomes, such as preterm birth,^47^ as was seen in our analysis.

In conclusion, this systematic review and meta-analysis found no evidence of adverse perinatal outcomes associated with oral PrEP or the dapivirine ring, compared to no PrEP or other PrEP modalities. There is low certainty of these findings because of the limited available data and low quality of the studies. For many PrEP modalities and perinatal outcomes assessed, there is a lack of evidence of harm, rather than evidence of lack of harm. However, the current data are reassuring, which support the WHO recommendation that TDF-based oral PrEP is safe during pregnancy. More data is needed to assess the safety of the dapivirine ring and CAB-LA.^10^ With the expansion of PrEP programmes and with more modalities of PrEP in development, it is imperative for researchers to include pregnant women in studies and to collect accurate and complete data on adverse perinatal outcomes.^48^ PrEP studies should pay particular attention to pregnancy outcomes which have been implicated by the use of the same antiretroviral drugs in people living with HIV, such as fetal growth/bone outcomes in relation to TDF.^40^ More research is needed to determine the safety profiles of different modalities of PrEP to give women of reproductive age and pregnant women in high HIV prevalence settings adequate tools to safeguard their own health and the health of their babies.

## Contributors

AE and IR wrote the study protocol, screened the electronic literature search results for relevant manuscripts, assessed their eligibility, extracted data, collected additional unpublished data, and made tables.

AE analysed and interpreted data, developed the figures, and wrote the first full draft of the manuscript.

SK designed and performed the electronic literature search.

JH conceived, designed and coordinated the study, wrote the study protocol, assisted with the literature search, assessed eligibility of manuscripts, collected additional unpublished data, designed the analysis, figures and tables, interpreted the data, and wrote the manuscript.

Authors AE, IR and JH had full access to and verified the data. All authors accept responsibility to submit the manuscript for publication.

## Data sharing statement

Study data are available on reasonable request to the corresponding author. The protocol for this review is available on the PROSPERO website (CRD42022339825).

## Declaration of interests

JH is an unpaid member of the HIV in pregnancy guideline committee of the British HIV Association (BHIVA). All other authors declare no competing interests.

## Appendix A Supplementary data

Appendix-PrEP-Final
