# Supplementary material for: Perinatal outcomes associated with pre-exposure prophylaxis for HIV prevention during pregnancy: a systematic review and meta-analysis
Source: eClinicalMedicine. 2024 Mar 19;70:102532. doi: 10.1016/j.eclinm.2024.102532 (PMC11056414; doi:10.1016/j.eclinm.2024.102532)
Supplement: Appendix-PrEP-Final [file mmc1.docx]

**Perinatal outcomes associated with pre-exposure prophylaxis for HIV prevention during pregnancy: a systematic review and meta-analysis.**

**APPENDICES**

[APPENDIX 1: Prisma checklist 4](#_Toc150727244)

[APPENDIX 2: Literature search strategies 8](#_Toc150727245)

[APPENDIX 3: Risk of bias assessment of randomised controlled trials (RCTs) 19](#_Toc150727246)

[Appendix 3.1: Cochrane RCT Risk of Bias (RoB 2) assessment tool 19](#_Toc150727247)

[Appendix 3.2: Risk of bias of RCTs included in the systematic review and meta-analysis 21](#_Toc150727248)

[APPENDIX 4: Quality assessment of cohort studies 22](#_Toc150727249)

[Appendix 4.1: Adapted Newcastle-Ottawa quality assessment tool 22](#_Toc150727250)

[Appendix 4.2: Classification of studies according to quality assessment 24](#_Toc150727251)

[Appendix 4.3: Quality assessment of cohort studies included in the systematic review and meta-analysis 25](#_Toc150727252)

[Appendix 4.4: Confounding factors adjusted for in included cohort studies 27](#_Toc150727253)

[APPENDIX 5: Fixed-effect meta-analyses of the association between PrEP exposure during pregnancy and adverse perinatal and maternal outcomes 29](#_Toc150727254)

[Appendix 5.1: Cohort studies assessing the association between low birthweight (LBW) and oral PrEP exposure compared to no drug 29](#_Toc150727255)

[Appendix 5.2: Cohort studies assessing the association between small for gestational age (SGA) and oral PrEP exposure compared to no drug 30](#_Toc150727256)

[Appendix 5.3: RCTs assessing the association between preterm birth (PTB) and dapivirine ring exposure compared to placebo 31](#_Toc150727257)

[Appendix 5.4: RCTs assessing preterm birth (PTB) and exposure to TDF/FTC oral pill compared to TDF oral pill. 32](#_Toc150727258)

[Appendix 6: Summary of outcomes from included studies 33](#_Toc150727259)

[Appendix 6.1: Odds ratios 33](#_Toc150727260)

[Appendix 6.2: Number of studies 36](#_Toc150727261)

[Appendix 6.3: Number of women analysed 39](#_Toc150727262)

[APPENDIX 7: Funnel plots to assess for small study effects 42](#_Toc150727263)

[Appendix 7.1: RCTs assessing the association between preterm birth (PTB) and oral PrEP exposure during pregnancy vs no oral PrEP 42](#_Toc150727264)

[Appendix 7.2: Cohort studies assessing the association between preterm birth (PTB) and oral PrEP exposure 43](#_Toc150727265)

[Appendix 7.3: Cohort studies assessing the association between low birth weight (LBW) and oral PrEP exposure 44](#_Toc150727266)

[Appendix 7.4: Cohort studies assessing the association between small for gestational age and oral PrEP exposure during pregnancy vs no PrEP exposure 45](#_Toc150727267)

[Appendix 7.5: RCTs assessing the association between preterm birth and dapivirine ring exposure compared to no dapivirine ring 46](#_Toc150727268)

[Appendix 7.6: RCTs assessing the association between preterm birth and TDF/FTC oral pill compared to TDF oral pill 47](#_Toc150727269)

[APPENDIX 8: Random effects meta-analyses of the association between PrEP exposure during pregnancy and adverse perinatal and maternal outcomes. 48](#_Toc150727270)

[Appendix 8.1: RCTs assessing the association between preterm birth (PTB) and oral PrEP exposure 48](#_Toc150727271)

[Appendix 8.2: Cohort studies assessing the association between preterm birth (PTB) and oral PrEP exposure 49](#_Toc150727272)

[Appendix 8.3: Cohort studies assessing the association between low birthweight (LBW) and oral PrEP exposure 50](#_Toc150727273)

[Appendix 8.4: Cohort studies assessing the association between small for gestational age (SGA) and oral PrEP exposure 51](#_Toc150727274)

[Appendix 8.5: RCTs assessing the association between preterm birth (PTB) and dapivirine ring exposure 52](#_Toc150727275)

# APPENDIX 1: Prisma checklist

| **Section/topic** | **#** | **Checklist item** | **Reported on page #** |
| --- | --- | --- | --- |
| **TITLE** | | |  |
| Title | 1 | Identify the report as a systematic review, meta-analysis, or both. | p1 |
| **ABSTRACT** | | |  |
| Structured summary | 2 | Provide a structured summary including, as applicable: background; objectives; data sources; study eligibility criteria, participants, and interventions; study appraisal and synthesis methods; results; limitations; conclusions and implications of key findings; systematic review registration number. | p2-3 |
| **INTRODUCTION** | | |  |
| Rationale | 3 | Describe the rationale for the review in the context of what is already known. | p5 |
| Objectives | 4 | Provide an explicit statement of questions being addressed with reference to participants, interventions, comparisons, outcomes, and study design (PICOS). | p5 |
| **METHODS** | | |  |
| Protocol and registration | 5 | Indicate if a review protocol exists, if and where it can be accessed (e.g., Web address), and, if available, provide registration information including registration number. | p6 |
| Eligibility criteria | 6 | Specify study characteristics (e.g., PICOS, length of follow-up) and report characteristics (e.g., years considered, language, publication status) used as criteria for eligibility, giving rationale. | p6 |
| Information sources | 7 | Describe all information sources (e.g., databases with dates of coverage, contact with study authors to identify additional studies) in the search and date last searched. | p6,p8 |
| Search | 8 | Present full electronic search strategy for at least one database, including any limits used, such that it could be repeated. | Appendix 2 |
| Study selection | 9 | State the process for selecting studies (i.e., screening, eligibility, included in systematic review, and, if applicable, included in the meta-analysis). | p7 |
| Data collection process | 10 | Describe method of data extraction from reports (e.g., piloted forms, independently, in duplicate) and any processes for obtaining and confirming data from investigators. | p8 |
| Data items | 11 | List and define all variables for which data were sought (e.g., PICOS, funding sources) and any assumptions and simplifications made. | p7 |
| Risk of bias in individual studies | 12 | Describe methods used for assessing risk of bias of individual studies (including specification of whether this was done at the study or outcome level), and how this information is to be used in any data synthesis. | p8 |
| Summary measures | 13 | State the principal summary measures (e.g., risk ratio, difference in means). | p9 |
| Synthesis of results | 14 | Describe the methods of handling data and combining results of studies, if done, including measures of consistency (e.g., I^2^) for each meta-analysis. | p9 |

| **Section/topic** | **#** | **Checklist item** | **Reported on page #** |
| --- | --- | --- | --- |
| Risk of bias across studies | 15 | Specify any assessment of risk of bias that may affect the cumulative evidence (e.g., publication bias, selective reporting within studies). | p9 |
| Additional analyses | 16 | Describe methods of additional analyses (e.g., sensitivity or subgroup analyses, meta-regression), if done, indicating which were pre-specified. | p9 |
| **RESULTS** | | |  |
| Study selection | 17 | Give numbers of studies screened, assessed for eligibility, and included in the review, with reasons for exclusions at each stage, ideally with a flow diagram. | p10  Figure 1 |
| Study characteristics | 18 | For each study, present characteristics for which data were extracted (e.g., study size, PICOS, follow-up period) and provide the citations. | p10, Table 1 |
| Risk of bias within studies | 19 | Present data on risk of bias of each study and, if available, any outcome level assessment (see item 12). | Table 1 |
| Results of individual studies | 20 | For all outcomes considered (benefits or harms), present, for each study: (a) simple summary data for each intervention group (b) effect estimates and confidence intervals, ideally with a forest plot. | Figure 2, Appendices 5 and 6 |
| Synthesis of results | 21 | Present results of each meta-analysis done, including confidence intervals and measures of consistency. | Figures 2-4, Appendix 5 |
| Risk of bias across studies | 22 | Present results of any assessment of risk of bias across studies (see Item 15). | p10 |
| Additional analysis | 23 | Give results of additional analyses, if done (e.g., sensitivity or subgroup analyses, meta-regression [see Item 16]). | N/A |
| **DISCUSSION** | | |  |
| Summary of evidence | 24 | Summarize the main findings including the strength of evidence for each main outcome; consider their relevance to key groups (e.g., healthcare providers, users, and policy makers). | p14,p17 |
| Limitations | 25 | Discuss limitations at study and outcome level (e.g., risk of bias), and at review-level (e.g., incomplete retrieval of identified research, reporting bias). | p16-17 |
| Conclusions | 26 | Provide a general interpretation of the results in the context of other evidence, and implications for future research. | p17 |
| **FUNDING** | | |  |
| Funding | 27 | Describe sources of funding for the systematic review and other support (e.g., supply of data); role of funders for the systematic review. | p9 |

# APPENDIX 2: Literature search strategies

| **MEDLINE**  **Database and platform: Medline (Ovid MEDLINE® Epub Ahead of Print, In-Process & Other Non-Indexed Citations, Ovid MEDLINE® Daily and Ovid MEDLINE®) 1946 to present**  **Latest search date: 29 August 2023** | |
| --- | --- |
| 1. Pregnancy Outcome/ or exp Pregnancy Complications, Infectious/  2. ((pregnancy or gestational or fetal or foetal or obstetric$) adj1 (outcome$ or complication$ or consequence$ or characteristic$ or event$ or result$ or problem$ or morbidit$ or sequelae)).ti,ab.  3. ((labor or labour or birth or delivery or neonate or newborn or "new-born" or "new born") adj1 (outcome$ or complication$ or consequence$ or characteristic$ or event$ or result$ or problem$ or morbidit$ or sequelae)).ti,ab.  4. ((infant or reproductive or prelabour or prelabor or "pre-labour" or "pre-labor" or intrauterine or "intra-uterine") adj1 (outcome$ or complication$ or consequence$ or characteristic$ or event$ or result$ or problem$ or morbidit$ or sequelae)).ti,ab.  5. ((antenatal or "ante-natal" or prenatal or "pre-natal" or perinatal or "peri-natal" or neonatal or "neo-natal" or postnatal or "post-natal") adj1 (outcome$ or complication$ or consequence$ or characteristic$ or event$ or result$ or problem$ or morbidit$ or sequelae)).ti,ab.  6. ((antepartum or "ante-partum" or intrapartum or "intra-partum" or peripartum or "peri-partum" or postpartum or "post-partum") adj1 (outcome$ or complication$ or consequence$ or characteristic$ or event$ or result$ or problem$ or morbidit$ or sequelae)).ti,ab.  7. Premature Birth/ or exp Fetal Membranes, Premature Rupture/ or Obstetric Labor, Premature/ or Infant, Extremely Premature/ or Infant, Premature/  8. (prematurity or "gestational age at birth" or "gestational age at delivery" or PTB or PTBs or VPTB or VPTBs or "pre-terms" or preterms or PTL or PTLs or VPTL or VPTLs or PTD or PTDs or VPTD or VPTDs or PROM or PPROM).ti,ab.  9. (("pre-term" or preterm or premature) adj2 (labour$ or labor$ or infant or deliver$ or birth$)).ti,ab.  10. ((preterm or "pre-term" or premature) adj1 rupture adj3 membrane$).ti,ab.  11. Fetal Growth Retardation/ or Infant, Low Birth Weight/ or Infant, Very Low Birth Weight/ or Infant, Extremely Low Birth Weight/ or Infant, Small for Gestational Age/  12. ((intrauterine or "intra-uterine" or fetal or foetal) adj1 growth adj1 (restrict$ or retardation)).ti,ab.  13. (SGA or SFGA or IUGR or FGR or "small for gestational age" or "small-for-gestational-age" or "small-for-gestational age" or "small for gestation" or "small-for-gestation").ti,ab.  14. (VSGA or "very-small-for-gestational-age" or "very-small-for-gestational age" or SFD or "small for dates" or "small-for-dates" or "weight for dates" or "weight for gestational age" or "weight for age at delivery" or "weight at delivery").ti,ab.  15. ("birthweight for dates" or "birthweight for gestational age" or "birthweight for age at delivery" or "birth weight for dates" or "birth weight for gestational age" or "birth weight for age at delivery" or "birth-weight for dates" or "birth-weight for gestational age" or "birth-weight for age at delivery").ti,ab.  16. (LBW or "low BW" or "low birth weight" or "low birth-weight" or "low-birth weight" or "low-birth-weight" or "low birthweight" or "low-birthweight" or "lower BW" or "lower birth weight" or "lower birth-weight" or "lower-birth weight" or "lower-birth-weight" or "lower birthweight" or "lower-birthweight").ti,ab.  17. ("reduced birth weight" or "reduced birthweight" or "reduced birth-weight" or VLBW or "very-low birthweight" or "very-low birth weight" or "very-low birth-weight" or "very-low-birthweight" or "very-low-birth-weight" or ELBW or "extremely-low birthweight" or "extremely-low birth weight" or "extremely-low birth-weight" or "extremely-low-birthweight" or "extremely-low-birth-weight").ti,ab. | 18. Stillbirth/ or Fetal Death/  19. (stillbirth$ or "still birth$" or stillborn$ or "still born$" or abortion$ or miscarriage$).ti,ab.  20. ((pregnancy or gestational or fetal or foetal or obstetric$ or labor or labour or birth) adj1 (death$ or loss$ or demise$ mortalit$)).ti,ab.  21. ((delivery or neonate or newborn or "new-born" or "new born" or infant or reproductive or prelabour or prelabor or "pre-labour" or "pre-labor") adj1 (death$ or loss$ or demise$ mortalit$)).ti,ab.  22. ((intrauterine or "intra-uterine" or antenatal or ante-natal or prenatal or "pre-natal" or perinatal or "peri-natal" or neonatal or "neo-natal" or postnatal or "post-natal" or antepartum or "ante-partum" or intrapartum or "intra-partum" or peripartum or "peri-partum" or postpartum or "post-partum") adj1 (death$ or loss$ or demise$ mortalit$)).ti,ab.  23. 1 or 2 or 3 or 4 or 5 or 6 or 7 or 8 or 9 or 10 or 11 or 12 or 13 or 14 or 15 or 16 or 17 or 18 or 19 or 20 or 21 or 22  24. Pre-exposure prophylaxis/ or Tenofovir/ or Emtricitabine/ or Lamivudine/ or Rilpivirine/ or Integrase inhibitors/ or Transdermal Patch/ or Anti-HIV Agents/ or Anti-Retroviral Agents/ or Anti-Infective Agents/ or "Vaginal Creams, Foams, and Jellies"/ or Reverse transcriptase inhibitors/  25. ("pre-exposure prophylaxis" or "preexposure prophylaxis" or "pre-exposure chemoprophylaxis" or "preexposure chemoprophylaxis" or "antiretroviral chemoprophylaxis" or "antiretroviral prophylaxis" or "anti-retroviral chemoprophylaxis" or "anti-retroviral prophylaxis" or "HIV chemoprophylaxis" or "HIV prophylaxis" or PrEP).ti,ab.  26. (Tenofovir or TFV or "Tenofovir disoproxil fumarate" or TDF or "Tenofovir diphosphate" or "TFV-DP" or "Tenofovir alafenamide" or TAF or Emtricitabine or FTC or Lamivudine or 3TC or "Tenofovir disoproxil fumarate plus emtricitabine" or "TDF-FTC" or "Tenofovir alafenamide plus emtricitabine" or "TAF/FTC" or "Tenofovir disoproxil fumarate/Lamivudine" or "TDF-3TC" or "TAF-3TC" or "Emtricitabine plus tenofovir disoproxil fumarate" or "FTC-TDF" or "TFV/DP" or "TDF/FTC" or "TAF-FTC" or "TAF/3TC" or "TDF/3TC" or "CAB/LA" or "RPV/LA" or "FTC/TDF").ti,ab.  27. ("vaginal microbicide" or "vaginal gel" or "microbicide gel" or microbicides or BufferGel or PRO2000 or "PRO2000/5" or Dapivirine or DPV or DAP or Vicriviroc or VCV or VVC or Cabotegravir or CAB or "CAB-LA" or Rilpivirine or RPV or "RPV-LA").ti,ab.  28. ("nonnucleoside reverse transcriptase inhibitor$" or "non-nucleoside reverse transcriptase inhibitor$" or NNRTI or "integrase inhibitor$" or "integrase strand transfer inhibitor$" or INSTI or "nucleoside reverse transcriptase translocation inhibitor$" or NRTTI or Lenacapavir or LEN or "GS-6207" or "capsid inhibitor" or Islatravir or ISL or "MK-8591" or "vaginal patch" or "sub-dermal implant" or "subdermal implant" or "vaginal film" or "vaginal insert" or "Griffithsin plus carrageenan fast-dissolve insert" or "MucoCept lactobacillus vaginal tablet" or Elvitegravir or EVG or "prototype extended-release elvitegravir osmotic insert" or "nucleoside reverse transcriptase inhibitor$" or "reverse transcriptase inhibitor$" or NRTI or "anti-HIV agent$" or "antiHIV agent$" or "anti-infective agent$" or "antiinfective agent$" or "vaginal cream$" or "vaginal foam$" or "vaginal jell$").ti,ab.  29. 24 or 25 or 26 or 27 or 28  30 23 and 29  31. limit 30 to yr="2000-2023" |
| **CINAHL**  **Database and platform: CINAHL (via EBSCOhost)**  **Latest search date: 29 August 2023** | |
| 1. MH Pregnancy Outcomes OR MW Pregnancy Outcomes OR MH Pregnancy Complications, Infectious OR MW Pregnancy Complications, Infectious  2. (TI (pregnancy OR gestational OR fetal OR foetal OR obstetric* OR labor OR labour OR birth OR delivery) N1 (outcome* OR complication* OR consequence* OR characteristic* OR event* OR result* OR problem* OR morbidit* OR sequelae) OR (AB (pregnancy OR gestational OR fetal OR foetal OR obstetric* OR labor OR labour OR birth OR delivery) N1 (outcome* OR complication* OR consequence* OR characteristic* OR event* OR result* OR problem* OR morbidit* OR sequelae))    3. (TI (neonate OR newborn OR "new-born" OR "new born" OR infant OR reproductive OR prelabour OR prelabor OR "pre-labour" OR "pre-labor") N1 (outcome* OR complication* OR consequence* OR characteristic* OR event* OR result* OR problem* OR morbidit* OR sequelae) OR (AB (neonate OR newborn OR "new-born" OR "new born" OR infant OR reproductive OR prelabour OR prelabor OR "pre-labour" OR "pre-labor") N1 (outcome* OR complication* OR consequence* OR characteristic* OR event* OR result* OR problem* OR morbidit* OR sequelae))  4. (TI (intrauterine OR "intra-uterine" OR antenatal OR "ante-natal" OR prenatal OR "pre-natal" OR perinatal OR "peri-natal" OR neonatal OR "neo-natal") N1 (outcome* OR complication* OR consequence* OR characteristic* OR event* OR result* OR problem* OR morbidit* OR sequelae) OR (AB (intrauterine OR "intra-uterine" OR antenatal OR "ante-natal" OR prenatal OR "pre-natal" OR perinatal OR "peri-natal" OR neonatal OR "neo-natal") N1 (outcome* OR complication* OR consequence* OR characteristic* OR event* OR result* OR problem* OR morbidit* OR sequelae))    5. (TI (postnatal OR "post-natal" OR antepartum OR "ante-partum" OR intrapartum OR "intra-partum" OR peripartum OR "peri-partum" OR postpartum OR "post-partum") N1 (outcome* OR complication* OR consequence* OR characteristic* OR event* OR result* OR problem* OR morbidit* OR sequelae) OR (AB (postnatal OR "post-natal" OR antepartum OR "ante-partum" OR intrapartum OR "intra-partum" OR peripartum OR "peri-partum" OR postpartum OR "post-partum") N1 (outcome* OR complication* OR consequence* OR characteristic* OR event* OR result* OR problem* OR morbidit* OR sequelae))  6. MH Childbirth, Premature OR MW Childbirth, Premature OR MH Fetal Membranes, Premature Rupture OR MW Fetal Membranes, Premature Rupture OR MH Infant, Premature OR MW Infant, Premature OR MH Labor, Premature OR MW Labor, Premature OR MH Outcomes of Prematurity OR MW Outcomes of Prematurity  7. ((TI prematurity OR "gestational age at birth" OR "gestational age at delivery" OR PTB OR PTBs OR VPTB OR VPTBs OR "pre-terms" OR preterms OR PTL OR PTLs OR VPTL OR VPTLs) OR (AB prematurity OR "gestational age at birth" OR "gestational age at delivery" OR PTB OR PTBs OR VPTB OR VPTBs OR "pre-terms" OR preterms OR PTL OR PTLs OR VPTL OR VPTLs))  8. (TI (preterm OR "pre-term" OR premature) N2 (labor* OR labour* OR birth OR deliver* OR infant) OR (AB (preterm OR "pre-term" OR premature) N2 (labor* OR labour* OR birth OR deliver* OR infant))  9. ((TI PTD OR PTDs OR VPTD OR VPTDs OR PROM OR PPROM) OR (AB PTD OR PTDs OR VPTD OR VPTDs OR PROM OR PPROM))  10. (TI (preterm OR "pre-term" OR premature) N1 ("rupture of membrane*" OR "rupture of fetal membrane*" OR "rupture of foetal membrane*") OR (AB (preterm OR "pre-term" OR premature) N1 ("rupture of membrane*" OR "rupture of fetal membrane*" OR "rupture of foetal membrane*"))  11. MH Fetal Growth Retardation OR MW Fetal Growth Retardation OR MH Infant, Low Birth Weight OR MW Infant, Low Birth Weight OR MH Infant, Very Low Birth Weight OR MW Infant, Very Low Birth Weight OR MH Infant, Small for Gestational Age OR MW Infant, Small for Gestational Age  12. (TI (intrauterine OR "intra-uterine" OR fetal OR foetal) N2 (restriction OR restricted OR retardation) OR (AB (intrauterine OR "intra-uterine" OR fetal OR foetal) N2 (restriction OR restricted OR retardation))  13. ((TI IUGR OR FGR OR SGA OR SFGA OR VSGA OR SFD OR LBW OR VLBW OR ELBW OR "small for gestational age" OR "small-for-gestational-age" OR "small-for-gestational age" OR "small for gestation" OR "small-for-gestation" OR "very-small-for-gestational-age" OR "very-small-for-gestational age" OR "small for dates" OR "small-for-dates" OR "weight for dates" OR "weight for gestational age" OR "weight for age at delivery" OR "weight at delivery") OR (AB IUGR OR FGR OR SGA OR SFGA OR VSGA OR SFD OR LBW OR VLBW OR ELBW OR "small for gestational age" OR "small-for-gestational-age" OR "small-for-gestational age" OR "small for gestation" OR "small-for-gestation" OR "very-small-for-gestational-age" OR "very-small-for-gestational age" OR "small for dates" OR "small-for-dates" OR "weight for dates" OR "weight for gestational age" OR "weight for age at delivery" OR "weight at delivery"))  14. ((TI "birthweight for dates" OR "birthweight for gestational age" OR "birthweight for age at delivery" OR "birth weight for dates" OR "birth weight for gestational age" OR "birth weight for age at delivery" OR "birth-weight for dates" OR "birth-weight for gestational age" OR "birth-weight for age at delivery") OR (AB "birthweight for dates" OR "birthweight for gestational age" OR "birthweight for age at delivery" OR "birth weight for dates" OR "birth weight for gestational age" OR "birth weight for age at delivery" OR "birth-weight for dates" OR "birth-weight for gestational age" OR "birth-weight for age at delivery"))  15. ((TI "low BW" OR "low birth weight" OR "low birth-weight" OR "low-birth weight" OR "low-birth-weight" OR "low birthweight" OR "low-birthweight" OR "lower BW" OR "lower birth weight" OR "lower birth-weight" OR "lower-birth weight" OR "lower-birth-weight" OR "lower birthweight" OR "lower-birthweight") OR (AB "low BW" OR "low birth weight" OR "low birth-weight" OR "low-birth weight" OR "low-birth-weight" OR "low birthweight" OR "low-birthweight" OR "lower BW" OR "lower birth weight" OR "lower birth-weight" OR "lower-birth weight" OR "lower-birth-weight" OR "lower birthweight" OR "lower-birthweight"))  16. ((TI "reduced birth weight" OR "reduced birthweight" OR "reduced birth-weight" OR "very-low birthweight" OR "very-low birth weight" OR "very-low birth-weight" OR "very-low-birthweight" OR "very-low-birth-weight" OR "extremely-low birthweight" OR "extremely-low birth weight" OR "extremely-low birth-weight" OR "extremely-low-birthweight" OR "extremely-low-birth-weight") OR (AB "reduced birth weight" OR "reduced birthweight" OR "reduced birth-weight" OR "very-low birthweight" OR "very-low birth weight" OR "very-low birth-weight" OR "very-low-birthweight" OR "very-low-birth-weight" OR "extremely-low birthweight" OR "extremely-low birth weight" OR "extremely-low birth-weight" OR "extremely-low-birthweight" OR "extremely-low-birth-weight")) | 17. MH Perinatal Death OR MW Perinatal Death  18. ((TI stillbirth* OR "still birth*" OR stillborn* OR "still born*" OR abortion* OR miscarriage*) OR (AB stillbirth* OR "still birth*" OR stillborn* OR "still born*" OR abortion* OR miscarriage*))  19. (TI (pregnancy OR gestation* OR fetal OR foetal OR obstetric* OR labor OR labour OR birth OR delivery OR neonate OR newborn OR "new-born" OR "new born" OR infant OR reproductive OR prelabour OR prelabor OR "pre-labour" OR "pre-labor") N1 (death* OR loss* OR demise* OR mortalit*) OR (AB (pregnancy OR gestation* OR fetal OR foetal OR obstetric* OR labor OR labour OR birth OR delivery OR neonate OR newborn OR "new-born" OR "new born" OR infant OR reproductive OR prelabour OR prelabor OR "pre-labour" OR "pre-labor") N1 (death* OR loss* OR demise* OR mortalit*))  20. (TI (intrauterine OR "intra-uterine" OR antenatal OR "ante-natal" OR prenatal OR "pre-natal" OR perinatal OR "peri-natal" OR neonatal OR "neo-natal" OR postnatal OR "post-natal") N1 (death* OR loss* OR demise* OR mortalit*) OR (AB (intrauterine OR "intra-uterine" OR antenatal OR "ante-natal" OR prenatal OR "pre-natal" OR perinatal OR "peri-natal" OR neonatal OR "neo-natal" OR postnatal OR "post-natal") N1 (death* OR loss* OR demise* OR mortalit*))  21. (TI (antepartum OR "ante-partum" OR intrapartum OR "intra-partum" OR peripartum OR "peri-partum" OR postpartum OR "post-partum") N1 (death* OR loss* OR demise* OR mortalit*) OR (AB (antepartum OR "ante-partum" OR intrapartum OR "intra-partum" OR peripartum OR "peri-partum" OR postpartum OR "post-partum") N1 (death* OR loss* OR demise* OR mortalit*))    22. S1 OR S2 OR S3 OR S4 OR S5 OR S6 OR S7 OR S8 OR S9 OR S10 OR S11 OR S12 OR S13 OR S14 OR S15 OR S16 OR S17 OR S18 OR S19 OR S20 OR S21  23. MH Pre-Exposure Prophylaxis OR MW Pre-Exposure Prophylaxis OR MH Tenofovir OR MW Tenofovir OR MH Emtricitabine OR MW Emtricitabine OR MH Lamivudine OR MW Lamivudine OR MH Emtricitabine Tenofovir MW Emtricitabine Tenofovir OR MH Rilpivirine OR MW Rilpivirine OR MH Non-Nucleoside Reverse Transcriptase Inhibitors OR MW Non-Nucleoside Reverse Transcriptase Inhibitors OR MH HIV Integrase Inhibitors OR MW HIV Integrase Inhibitors OR MH Anti-HIV Agents OR MW Anti-HIV Agents OR MH Vaginal Creams, Foams and Jellies OR MW Vaginal Creams, Foams and Jellies OR MH Antiinfective Agents OR MW Antiinfective Agents OR MH Nucleoside Reverse Transcriptase Inhibitors OR MW Nucleoside Reverse Transcriptase Inhibitors OR MH Reverse Transcriptase Inhibitors OR MW Reverse Transcriptase Inhibitors  24. ((TI "pre-exposure prophylaxis" OR "preexposure prophylaxis" OR "pre-exposure chemoprophylaxis" OR "preexposure chemoprophylaxis" OR "antiretroviral chemoprophylaxis" OR "antiretroviral prophylaxis" OR "anti-retroviral chemoprophylaxis" OR "anti-retroviral prophylaxis" OR "HIV chemoprophylaxis" OR "HIV prophylaxis" OR PrEP OR "anti-HIV agent*" OR "antiHIV agent*" OR "anti-infective agent*" OR "antiinfective agent*" OR "vaginal cream*" OR "vaginal foam*" OR "vaginal jell*") OR (AB "pre-exposure prophylaxis" OR "preexposure prophylaxis" OR "pre-exposure chemoprophylaxis" OR "preexposure chemoprophylaxis" OR "antiretroviral chemoprophylaxis" OR "antiretroviral prophylaxis" OR "anti-retroviral chemoprophylaxis" OR "anti-retroviral prophylaxis" OR "HIV chemoprophylaxis" OR "HIV prophylaxis" OR PrEP OR "anti-HIV agent*" OR "antiHIV agent*" OR "anti-infective agent*" OR "antiinfective agent*" OR "vaginal cream*" OR "vaginal foam*" OR "vaginal jell*"))  25. ((TI Tenofovir OR TFV OR "Tenofovir disoproxil fumarate" OR TDF OR "Tenofovir diphosphate" OR "TFV-DP" OR "Tenofovir alafenamide" OR TAF OR Emtricitabine OR FTC OR Lamivudine OR 3TC OR "Tenofovir disoproxil fumarate plus emtricitabine" OR "TDF-FTC" OR "Tenofovir alafenamide plus emtricitabine" OR "TAF/FTC" OR "Tenofovir disoproxil fumarate/Lamivudine" OR "TDF-3TC" OR "TAF-3TC" OR "Emtricitabine plus tenofovir disoproxil fumarate" OR "FTC-TDF" OR "TFV/DP" OR "TDF/FTC" OR "TAF-FTC" OR "TAF/3TC" OR "TDF/3TC" OR "CAB/LA" OR "RPV/LA" or "FTC/TDF") OR (AB Tenofovir OR TFV OR "Tenofovir disoproxil fumarate" OR TDF OR "Tenofovir diphosphate" OR "TFV-DP" OR "Tenofovir alafenamide" OR TAF OR Emtricitabine OR FTC OR Lamivudine or 3TC or "Tenofovir disoproxil fumarate plus emtricitabine" OR "TDF-FTC" OR "Tenofovir alafenamide plus emtricitabine" OR "TAF/FTC" OR "Tenofovir disoproxil fumarate/Lamivudine" OR "TDF-3TC" OR "TAF-3TC" OR "Emtricitabine plus tenofovir disoproxil fumarate" OR "FTC-TDF" OR "TFV/DP" OR "TDF/FTC" OR "TAF-FTC" OR "TAF/3TC" OR "TDF/3TC" OR "CAB/LA" OR "RPV/LA" or "FTC/TDF"))  26. ((TI "vaginal microbicide" OR "vaginal gel" OR "microbicide gel" OR microbicides OR BufferGel OR PRO2000 OR "PRO2000/5" OR Dapivirine OR DPV OR DAP OR Vicriviroc OR VCV OR VVC OR Cabotegravir OR CAB OR "CAB-LA" OR Rilpivirine OR RPV OR "RPV-LA") OR (AB "vaginal microbicide" OR "vaginal gel" OR "microbicide gel" OR microbicides OR BufferGel OR PRO2000 OR "PRO2000/5" OR Dapivirine OR DPV OR DAP OR Vicriviroc OR VCV OR VVC OR Cabotegravir OR CAB OR "CAB-LA" OR Rilpivirine OR RPV OR "RPV-LA"))  27. ((TI "nonnucleoside reverse transcriptase inhibitor*" OR "non-nucleoside reverse transcriptase inhibitor*" OR NNRTI OR "integrase inhibitor*" OR "integrase strand transfer inhibitor*" OR INSTI OR "nucleoside reverse transcriptase translocation inhibitor*" OR NRTTI OR Lenacapavir OR LEN OR "GS-6207" OR "capsid inhibitor" OR Islatravir OR ISL OR "MK-8591" OR "vaginal patch" OR "sub-dermal implant" OR "subdermal implant" OR "vaginal film" OR "vaginal insert" OR "Griffithsin plus carrageenan fast-dissolve insert" OR "MucoCept lactobacillus vaginal tablet" OR Elvitegravir OR EVG OR "prototype extended-release elvitegravir osmotic insert" OR "nucleoside reverse transcriptase inhibitor*" OR "reverse transcriptase inhibitor*" OR NRTI) OR (AB "nonnucleoside reverse transcriptase inhibitor*" OR "non-nucleoside reverse transcriptase inhibitor*" OR NNRTI OR "integrase inhibitor*" OR "integrase strand transfer inhibitor*" OR INSTI OR "nucleoside reverse transcriptase translocation inhibitor*" OR NRTTI OR Lenacapavir OR LEN OR "GS-6207" OR "capsid inhibitor" OR Islatravir OR ISL OR "MK-8591" OR "vaginal patch" OR "sub-dermal implant" OR "subdermal implant" OR "vaginal film" OR "vaginal insert" OR "Griffithsin plus carrageenan fast-dissolve insert" OR "MucoCept lactobacillus vaginal tablet" OR Elvitegravir OR EVG OR "prototype extended-release elvitegravir osmotic insert" OR "nucleoside reverse transcriptase inhibitor*" OR "reverse transcriptase inhibitor*" OR NRTI))  28. S23 OR S24 OR S25 OR S26 OR S27  29. S22 AND S28  30. DT 2000-2023  31. S29 AND S30 |
| **GLOBAL HEALTH**  **Database and platform: Global Health 1973 to 2023 Week 34 (via OVID)**  **Latest search date: 29 August 2023** | |
| 1. Pregnancy Complications/ or Parturition complications/  2. ((pregnancy or gestational or fetal or foetal or obstetric$ or labor or labour or birth or delivery or neonat$ or newborn or "new-born" or "new born") adj1 (outcome$ or complication$ or consequence$ or characteristic$ or event$ or result$ or problem$ or morbidit$ or sequelae)).ti,ab.  3. ((infant or reproductive or prelabour or prelabor or "pre-labour" or "pre-labor" or intrauterine or "intra-uterine" or antenatal or "ante-natal" or prenatal or "pre-natal" or perinatal or "peri-natal") adj1 (outcome$ or complication$ or consequence$ or characteristic$ or event$ or result$ or problem$ or morbidit$ or sequelae)).ti,ab.  4. ((neonatal or "neo-natal" or postnatal or "post-natal" or antepartum or "ante-partum" or intrapartum or "intra-partum" or peripartum or "peri-partum" or postpartum or "post-partum") adj1 (outcome$ or complication$ or consequence$ or characteristic$ or event$ or result$ or problem$ or morbidit$ or sequelae)).ti,ab.  5. Prematurity/ or Premature infants/ or Fetal membranes/  6. ("premature birth" or prematurity or "gestational age at birth" or "gestational age at delivery" or "pre-terms" or preterms).ti,ab.  7. (PTB or PTBS or VPTB or VPTBs or PTLs or VPTL or VPTLs or PTD or PTDs or VPTD or VPTDs or PROM or PPROM).ti,ab.  8. (("pre-term" or preterm or premature) adj1 (birth$ or labour$ or labor$ or deliver$ or infant$).ti,ab.  9. (("pre-term" or preterm or premature) adj3 obstetric adj3 (labor$ or labour$)).ti,ab.  10. (("pre-term" or preterm or premature) adj1 ("rupture of membranes" or "rupture of fetal membranes" or "rupture of foetal membranes")).ti,ab.  11. Growth retardation/ or Birth weight/ or Low birth weight infants/  12. (IUGR or FGR or SGA or SFGA or VSGA or SFD or LBW or VLBW or ELBW).ti,ab.  13. ((intrauterine or "intra-uterine" or fetal or foetal) adj1 growth adj1 (restriction or restricted or retardation)).ti,ab.  14. ("small for gestational age" or "small-for-gestational-age" or "small-for-gestational age" or "small for gestation" or "small-for-gestation" or "very-small-for-gestational-age" or "very-small-for-gestational age" or "small for dates" or "small-for-dates" or "weight for dates" or "weight for gestational age" or "weight for age at delivery" or "weight at delivery" or "birthweight for dates" or "birthweight for gestational age" or "birthweight for age at delivery" or "birth weight for dates" or "birth weight for gestational age" or "birth weight for age at delivery" or "birth-weight for dates" or "birth-weight for gestational age" or "birth-weight for age at delivery").ti,ab.  15. ("low BW" or "low birth weight" or "low birth-weight" or "low-birth weight" or "low-birth-weight" or "low birthweight" or "low-birthweight" or "lower BW" or "lower birth weight" or "lower birth-weight" or "lower-birth weight" or "lower-birth-weight" or "lower birthweight" or "lower-birthweight" or "reduced birth weight" or "reduced birthweight" or "reduced birth-weight" or "very-low birthweight" or "very-low birth weight" or "very-low birth-weight" or "very-low-birthweight" or "very-low-birth-weight" or "extremely-low birthweight" or "extremely-low birth weight" or "extremely-low birth-weight" or "extremely-low-birthweight" or "extremely-low-birth-weight").ti,ab.  16. Stillbirths/ or Fetal death/ | 17. (stillbirth$ or stillborn$ or abortion$ or miscarriage$).ti,ab.  18. (still adj1 (born$ or birth$)).ti,ab.  19. ((pregnancy or gestation$ or fetal or foetal or obstetric$ or labour or labor or birth or delivery or neonat$ or newborn or "new-born" or "new born" or infant or reproductive or prelabour or "pre-labour" or prelabor or "pre-labor" or intrauterine or "intra-uterine" or antenatal or "ante-natal" or prenatal or "pre-natal") adj1 (death$ or loss$ or demise$ or mortalit$)).ti,ab.  20. ((perinatal or "peri-natal" or "neo-natal" or postnatal or "post-natal" or antepartum or "ante-partum" or intrapartum or "intra-partum" or peripartum or "peri-partum" or postpartum or "post-partum") adj1 (death$ or loss$ or demise$ or mortalit$)).ti,ab.  21. 1 or 2 or 3 or 4 or 5 or 6 or 7 or 8 or 9 or 10 or 11 or 12 or 13 or 14 or 15 or 16 or 17 or 18 or 19 or 20  22. Tenofovir/ or Emtricitabine/ or Lamivudine/ or Microbicides/ or Rilpivirine/ or Elvitegravir/ or Prophylaxis/ or Chemoprophylaxis/ or Antiretroviral agents/ or Reverse transcriptase inhibitors/ or "Non-nucleoside reverse transcriptase inhibitors"/ or Antiinfective agents/  23. ("pre-exposure prophylaxis" or "preexposure prophylaxis" or "pre-exposure chemoprophylaxis" or "preexposure chemoprophylaxis" or "antiretroviral chemoprophylaxis" or "antiretroviral prophylaxis" or "anti-retroviral chemoprophylaxis" or "anti-retroviral prophylaxis" or "HIV chemoprophylaxis" or "HIV prophylaxis" or PrEP).ti,ab.  24. (Tenofovir or TFV or "Tenofovir disoproxil fumarate" or TDF or "Tenofovir diphosphate" or "TFV-DP" or "Tenofovir alafenamide" or TAF or Emtricitabine or FTC or Lamivudine or 3TC or "Tenofovir disoproxil fumarate plus emtricitabine" or "TDF-FTC" or "Tenofovir alafenamide plus emtricitabine" or "TAF/FTC" or "Tenofovir disoproxil fumarate/Lamivudine" or "TDF-3TC" or "TAF-3TC" or "Emtricitabine plus tenofovir disoproxil fumarate" or "FTC-TDF" or "TFV/DP" or "TDF/FTC" or "TAF-FTC" or "TAF/3TC" or "TDF/3TC" or "CAB/LA" or "RPV/LA" or "FTC/TDF").ti,ab.  25. ("vaginal microbicide" or "vaginal gel" or "microbicide gel" or microbicides or BufferGel or PRO2000 or "PRO2000/5" or Dapivirine or DPV or DAP or Vicriviroc or VCV or VVC or Cabotegravir or CAB or "CAB-LA" or Rilpivirine or RPV or "RPV-LA").ti,ab.  26. ("nonnucleoside reverse transcriptase inhibitor$" or "non-nucleoside reverse transcriptase inhibitor$" or NNRTI or "integrase inhibitor$" or "integrase strand transfer inhibitor$" or INSTI or "nucleoside reverse transcriptase translocation inhibito$" or NRTTI or Lenacapavir or LEN or "GS-6207" or "capsid inhibitor" or Islatravir or ISL or "MK-8591" or "vaginal patch" or "sub-dermal implant" or "subdermal implant" or "vaginal film" or "vaginal insert" or "Griffithsin plus carrageenan fast-dissolve insert" or "MucoCept lactobacillus vaginal tablet" or Elvitegravir or EVG or "prototype extended-release elvitegravir osmotic insert" or "nucleoside reverse transcriptase" or "reverse transcriptase inhibitor$" or NRTI or "anti-HIV agent$" or "antiHIV agent$" or "anti-infective agent$" or "antiinfective agent$"or "vaginal cream$" or "vaginal foam$" or "vaginal jell$").ti,ab.  27. 22 or 23 or 24 or 25 or 26  28. 21 and 27  29. limit 28 to yr="2000-2023" |
| **EMBASE**  **Database and platform: Embase 1974 to present (via OVID)**  **Latest search date: 29 August 2023** | |
| 1. Pregnancy outcome/ or Pregnancy Complication/ or Fetus outcome/ or Labor complication/ or Perinatal morbidity/  2. ((pregnancy or gestation$ or fetal or foetal or obstetric$ or labor or labour or birth or delivery or neonate or newborn or "new-born" or "new born") adj1 (outcome$ or complication$ or consequence$ or characteristic$ or event$ or result$ or problem$ or morbidit$ or sequelae)).ti,ab.  3. ((infant or reproductive or prelabour or prelabor or "pre-labour" or "pre-labor" or intrauterine or "intra-uterine" or antenatal or "ante-natal" or prenatal or "pre-natal") adj1 (outcome$ or complication$ or consequence$ or characteristic$ or event$ or result$ or problem$ or morbidit$ or sequelae)).ti,ab.  4. ((perinatal or "peri-natal" or neonatal or "neo-natal" or postnatal or "post-natal" or antepartum or "ante-partum" or intrapartum or "intra-partum" or peripartum or "peri-partum" or postpartum or "post-partum") adj1 (outcome* or complication* or consequence* or characteristic* or event* or result* or problem* or morbidit* or sequelae)).ti,ab.  5. Prematurity/ or Premature labor/ or Premature fetus membrane rupture/ or "Immature and premature labor"/  6. ("premature birth" or prematurity or "gestational age at birth" or "gestational age at delivery" or "pre-terms" or preterms).ti,ab.  7. (PTB or PTBs or VPTB or VPTBs or PTL or PTLs or VPTL or VPTLs or PTD or PTDs or VPTD or VPTDs or PROM or PPROM).ti,ab.  8. (("pre-term" or preterm or premature) adj1 (birth$ or labour$ or labor$ or deliver$ or infant$)).ti,ab.  9. (("pre-term" or preterm or premature) adj3 obstetric adj3 (labor$ or labour$)).ti,ab.  10. (("pre-term" or preterm or premature) adj1 ("rupture of membranes" or "rupture of fetal membranes" or "rupture of foetal membranes")).ti,ab.  11. Intrauterine growth retardation/ or Small for date infant/ or Low birth weight/ or Very low birthweight/ or Extremely low birth weight/  12. (IUGR or FGR or SGA or SFGA or VSGA or SFD or LBW or VLBW or ELBW).ti,ab.  13. ((intrauterine or "intra-uterine" or fetal or foetal) adj1 growth adj1 (restriction or restricted or retardation)).ti,ab.  14. ("small for gestational age" or "small-for-gestational-age" or "small-for-gestational age" or "small for gestation" or "small-for-gestation" or "very-small-for-gestational-age" or "very-small-for-gestational age" or "small for dates" or "small-for-dates" or "weight for dates" or "weight for gestational age" or "weight for age at delivery" or "weight at delivery" or "birthweight for dates" or "birthweight for gestational age" or "birthweight for age at delivery" or "birth weight for dates" or "birth weight for gestational age" or "birth weight for age at delivery" or "birth-weight for dates" or "birth-weight for gestational age" or "birth-weight for age at delivery").ti,ab.  15. ("low BW" or "low birth weight" or "low birth-weight" or "low-birth weight" or "low-birth-weight" or "low birthweight" or "low-birthweight" or "lower BW" or "lower birth weight" or "lower birth-weight" or "lower-birth weight" or "lower-birth-weight" or "lower birthweight" or "lower-birthweight" or "reduced birth weight" or "reduced birthweight" or "reduced birth-weight" or "very-low birthweight" or "very-low birth weight" or "very-low birth-weight" or "very-low-birthweight" or "very-low-birth-weight" or "extremely-low birthweight" or "extremely-low birth weight" or "extremely-low birth-weight" or "extremely-low-birthweight" or "extremely-low-birth-weight").ti,ab.  16. Stillbirth/ or Fetus death/ | 17. (stillbirth$ or stillborn$ or abortion$ or miscarriage$).ti,ab.    18. (still adj1 (born$ or birth$)).ti,ab.  19. ((pregnancy or gestation$ or fetal or foetal or obstetric$ or labour or labor or birth or delivery or neonat$ or newborn or "new-born" or "new born" or infant or reproductive or prelabour or "pre-labour" or prelabor or "pre-labor" or intrauterine or "intra-uterine" or antenatal or "ante-natal" or prenatal or "pre-natal") adj1 (death$ or loss$ or demise$ or mortalit$)).ti,ab.  20. ((perinatal or "peri-natal" or "neo-natal" or postnatal or "post-natal" or antepartum or "ante-partum" or intrapartum or "intra-partum" or peripartum or "peri-partum" or postpartum or "post-partum") adj1 (death$ or loss$ or demise$ or mortalit$)).ti,ab.  21. 1 or 2 or 3 or 4 or 5 or 6 or 7 or 8 or 9 or 10 or 11 or 12 or 13 or 14 or 15 or 16 or 17 or 18 or 19 or 20  22. Pre-exposure prophylaxis/ or Tenofovir/ or Tenofovir alafenamide/ or Emtricitabine/ or Lamivudine/ or PRO2000/ or Dapivirine/ or Vicriviroc/ or Cabotegravir/ or Rilpivirine/ or Nonnucleoside reverse transcriptase inhibitor/ or Integrase inhibitor/ or Lenacapavir/ or Islatravir/ or Transdermal Patch/ or Griffithsin/ or Elvitegravir/ or Chemoprophylaxis/ or Antiretrovirus agent/ or Tenofovir disoproxil/ or "emtricitabine plus tenofovir disoproxil"/ or "emtricitabine plus tenofovir alafenamide"/ or "lamivudine plus tenofovir disoproxil"/ or Microbicide/ or "cabotegravir plus rilpivirine"/ or "emtricitabine plus rilpivirine plus tenofovir disoproxil"/ or "anti human immunodeficiency virus agent"/ or Vaginal ring/ or "RNA directed DNA polymerase inhibitor"/ or Antiinfective agent/  23. ("pre-exposure prophylaxis" or "preexposure prophylaxis" or "pre-exposure chemoprophylaxis" or "preexposure chemoprophylaxis" or "antiretroviral chemoprophylaxis" or "antiretroviral prophylaxis" or "anti-retroviral chemoprophylaxis" or "anti-retroviral prophylaxis" or "HIV chemoprophylaxis" or "HIV prophylaxis" or PrEP).ti,ab.  24. (Tenofovir or TFV or "Tenofovir disoproxil fumarate" or TDF or "Tenofovir diphosphate" or "TFV-DP" or "Tenofovir alafenamide" or TAF or Emtricitabine or FTC or Lamivudine or 3TC or "Tenofovir disoproxil fumarate plus emtricitabine" or "TDF-FTC" or "Tenofovir alafenamide plus emtricitabine" or "TAF/FTC" or "Tenofovir disoproxil fumarate/Lamivudine" or "TDF-3TC" or "TAF-3TC" or "Emtricitabine plus tenofovir disoproxil fumarate" or "FTC-TDF" or "TFV/DP" or "TDF/FTC" or "TAF-FTC" or "TAF/3TC" or "TDF/3TC" or "CAB/LA" or "RPV/LA" or "FTC/TDF").ti,ab.  25. ("vaginal microbicide" or "vaginal gel" or "microbicide gel" or microbicides or BufferGel or PRO2000 or "PRO2000/5" or Dapivirine or DPV or DAP or Vicriviroc or VCV or VVC or Cabotegravir or CAB or "CAB-LA" or Rilpivirine or RPV or "RPV-LA").ti,ab.  26. ("nonnucleoside reverse transcriptase inhibitor$" or "non-nucleoside reverse transcriptase inhibitor$" or NNRTI or "integrase inhibitor$" or "integrase strand transfer inhibitor$" or INSTI or "nucleoside reverse transcriptase translocation inhibitor$" or NRTTI or Lenacapavir or LEN or "GS-6207" or "capsid inhibitor" or Islatravir or ISL or "MK-8591" or "vaginal patch" or "sub-dermal implant" or "subdermal implant" or "vaginal film" or "vaginal insert" or "Griffithsin plus carrageenan fast-dissolve insert" or "MucoCept lactobacillus vaginal tablet" or Elvitegravir or EVG or "prototype extended-release elvitegravir osmotic insert" or "nucleoside reverse transcriptase inhibitor$" or "reverse transcriptase inhibitor$" or NRTI or "anti-HIV agent$" or "antiHIV agent$" or "antiinfective agent$" or "anti-infective agent$" or "vaginal cream$" or "vaginal foam$" or "vaginal jell$").ti,ab.  27. 22 or 23 or 24 or 25 or 26  28. 21 and 27  29. Limit 28 to yr="2000-2023" |

| **Cochrane review**  Database and platform: Cochrane  Latest search date: 29 August 2023  Filter for “HIV” AND “infectious disease” from 1 Jan 2000 to 29 Aug 2023 |
| --- |
| **Clinical trials.gov**  Database and platform:  Latest search date: 29 August 2023  Filter for HIV AND prevention from 1 Jan 2000 to 29 Aug 2023 |
| **WHO International Clinical Trials**    Database and platform:  Latest search date: 29 August 2023  Filter for “HIV” condition from from 1 Jan 2000 to 29 Aug 2023 |
| **ISRCTN**  Database and platform:  Latest search date: 29 August 2023  Filter for “HIV” condition from from 1 Jan 2000 to 29 Aug 2023 |
| **Pan African Trials database**  Database and platform:  Latest search date: 29 August 2023  Filter for “HIV” from from 1 Jan 2000 to 29 Aug 2023 |

# APPENDIX 3: Risk of bias assessment of randomized controlled trials (RCTs)

## Appendix 3.1: Cochrane RCT Risk of Bias (RoB 2) assessment tool

| The Cochrane risk of bias tool assesses bias across 5 domains to determine an overall risk of bias: the randomisation process, deviations from the intended interventions, missing outcome data, measurement of the outcome, and selection of the reported results.  Signalling questions were used within each domain to elicit relevant information to assess bias. Responses to signalling questions were   1. Yes 2. Probably yes 3. Probably no 4. No 5. No information   A response of Yes or No indicate that there is firm evidence related to the question. Qualifying either response with ‘Probably’ indicates a judgement has been made by the quality assessor. ‘Yes’ or ‘Probably yes’ are treated the same for risk of bias determination, as are ‘No’ and ‘Probably no’. No information indicates that there are insufficient details reported to make a definitive determination or make a reasonable judgement.  The possible risk of bias judgements are 1) low risk of bias, 2) Some concerns, and 3) high risk of bias. | | |
| --- | --- | --- |
| Domain 1: Risk of bias arising from the randomization process  1.1. Was the allocation sequence random?  1.2. Was the allocation sequence concealed until participants were enrolled and assigned to interventions?  1.3. Did the baseline differences between intervention groups suggest a problem with the randomization process? | Domain 2: Risk of bias due to deviations from the intended interventions (effect of assignment to intervention)  2.1. Were the participants aware of their assigned intervention during the trial?  2.2. Were carers and people delivering the intervention aware of participants’ assigned intervention during the trial?  2.3. If ‘yes, probably yes, or no information’ to 2.1. or 2.2.: Were there deviations from the intended intervention that arose because of the trial context?  2.4. If ‘yes or probably yes’ to 2.3: Were these deviations likely to have affected the outcome?  2.5. If ‘yes, probably yes, or no information’ to 2.4: Were these deviations from intended intervention balanced between groups?  2.6. Was an appropriate analysis used to estimate the effect of assignment to intervention?  2.7. If ‘yes, probably yes, or no information’ to 2.6.: Was there potential for a substantial impact (on the result) of the failure to analyse participants in the group to which they were randomized? | |
| Domain 3: Missing outcome data  3.1. Were data for this outcome available for all, or nearly all, participants randomized?  3.2. If ‘no, probably no, or no information’: Is there evidence that the result was not biased by missing outcome data?  3.3. If ‘no or probably no’ to 3.2: Could missingness in the outcome depend on its true value?  3.4. If ‘yes, probably yes, or no information’: Is it likely that missingness in the outcome depended on its true value? | Domain 4: Risk of bias in measurement of the outcome  4.1. Was the method of measuring the outcome appropriate (i.e., first trimester ultrasound to determine gestational age?  4.2. Could measurement or ascertainment of the outcome have differed between intervention groups?  4.3. If ‘no, probably no, or no information’ to 4.1. and 4.2. Were outcome assessors aware of the intervention received by study participants?  4.4. If ‘yes’, probably yes, or no information’ to 4.3: Could assessment of the outcome have been influenced by knowledge of intervention received?  4.5. If ‘yes, probably yes, or no information’ to 4.4.: Is it likely that assessment of the outcome was influenced by knowledge of intervention received? | Domain 5: Risk of bias in selection of the reported result  5.1. Were the data that produced this result analysed in accordance with a pre-specified analysis plan that was finalised before unblinded outcome data were available for analysis?  Is numerical result being assessed likely to have been selected on the basis of the results from…  5.2. … multiple eligible outcome measurements (e.g. scales, definitions, time points) within the outcome domain?  5.3. … multiple eligible analyses of the data? |

## Appendix 3.2: Risk of bias of RCTs included in the systematic review and meta-analysis

| **Study** | **Randomisation process** | **Deviations from the intended interventions** | **Missing outcomes** | **Measurement of the outcome** | **Selection of reported results** | **Overall risk of bias** |
| --- | --- | --- | --- | --- | --- | --- |
| Bunge 2015 | Low | Low | Low | High | Low | High |
| Bunge 2023 | Low | Low | Low | High | Low | High |
| Callahan 2015 | Low | Low | Low | High | Low | High |
| Kusemererwa 2018 | Low | Low | Low | High | Low | High |
| Makanani 2018 | Low | Low | Low | High | Low | High |
| Moodley 2023 | Low | Low | Low | High | Low | High |
| Mugo 2014 | Low | Low | Low | High | Low | High |
| Nel 2016 | Low | Low | Low | High | Low | High |

# APPENDIX 4: Quality assessment of cohort studies

## Appendix 4.1: Adapted Newcastle-Ottawa quality assessment tool

| A study can be awarded a maximum of one point (for items indicated with an asterisk) for each numbered criterion within the “Selection” and “Outcome” categories. | | |
| --- | --- | --- |
| - Selection (maximum 4 points) - Representativeness of the exposed cohort   - Truly representative of the pregnant population in the community*   - Somewhat representative of the pregnant population in the community   - Selected group of users, e.g. nurses, volunteers, teenage mothers   - No description of the derivation of the cohort - Selection of the comparator cohort   - The comparator cohort is drawn from the same community as the exposed cohort*   - The comparator cohort is drawn from a different source than the exposed cohort   - No description of the derivation of the comparator cohort - Ascertainment of exposure   - PrEP intake monitored as part of the study*   - PrEP intake confirmed from secure medical records (e.g. hospital records)*   - Structured interview-participant reported PrEP intake   - Written self-report   - No description - Demonstration that outcome of interest was not present at start of study   - Yes*   - No | - Comparability (maximum 2 points) - Comparability of cohorts on the basis of the analysis. In the analysis:   - Study controls for BMI, smoking, parity, and maternal age*   - Study controls for one or more additional factors e.g. prior history of adverse pregnancy outcome, maternal hypertension, anaemia, illicit drug or alcohol use in pregnancy*   - Confounding factors not controlled for | - Outcome (maximum 3 points) - Ascertainment of outcome   - Outcome was confirmed following clinical observation of outcome by clinician, midwife or trained birth attendant*   - Medical records*   - Self-report   - No description - Method used to assess gestational age   - Gestational age was determined according to early ultrasound (<14 weeks)*   - Gestational age was determined by: late ultrasound (≥14 weeks’ gestation) or last normal menstrual period or neonatal assessment, e.g. Ballard score, or a combination of these methods   - No description - Follow-up of cohorts   - Complete follow-up – all subjects accounted for*   - Subjects lost to follow-up unlikely to introduce bias, i.e. <20% lost to follow-up*   - Follow-up rate <80% (lost to follow-up >20%)   - No description |

## Appendix 4.2: Classification of studies according to quality assessment

| Adapted Newcastle-Ottawa quality assessment tool for cohort studies | |
| --- | --- |
| Overall study quality | Criteria |
| Good Quality | All requirements met – 9 for cohort study or 10 for case control |
| Average Quality | 3 points in “Selection” and 3 points in “Outcome” sections |
|  | ≥2 points in the “Selection” and “Outcome” sections, as well as ≥1 point in the “Comparability” section. |
| Poor Quality | < 2 points in the “Selection” and/or “Outcome” sections. |
|  | 2 points in the “Selection” and “Outcome” sections, but no points in the “Comparability” section. |
| Adapted Cochrane risk of bias tool for individual randomised controlled trials | |
| Overall risk-of-bias judgement | Criteria |
| Low risk of bias | The study is judged to be at low risk of bias for all domains for this result |
| Some concerns | The study is judged to raise some concerns in at least one domain for this result, but not to be at high risk of bias for any domain |
| High risk of bias | The study is judged to be at high risk of bias in at least one domain for this result Or  The study is judged to have some concerns for multiple domains in a way that substantially lowers confidence in the result |

## Appendix 4.3: Quality assessment of cohort studies included in the systematic review and meta-analysis

|  | **SELECTION** | | | |  | **COMPARABILITY** | |  | **OUTCOME** | | |  |
| --- | --- | --- | --- | --- | --- | --- | --- | --- | --- | --- | --- | --- |
| **Study** | **Representativeness of the exposed cohort** | **Selection of comparator cohort** | **Ascertainment of exposure** | **Demonstration that outcome of interest was not present at start of study** |  | **Study controls for BMI, smoking, parity, and maternal age** | **Study controls for one or more additional factors** |  | **Ascertainment of outcome** | **Method used to assess gestational age** | **Follow-up cohorts** | **Total quality of assessment** |
| Davey 2022 | Truly representative of the pregnant population in the community* | The comparator cohort is drawn from the same community as the exposed cohort* | PrEP intake confirmed from secure medical records (e.g. hospital records)* | Yes* |  | No | No |  | Medical records* | No description | Complete follow - all subjects accounted for* | Poor |
| Dettinger 2019 | Somewhat representative of the pregnant population in the community | The comparator cohort is drawn from the same community as the exposed cohort* | PrEP intake confirmed from secure medical records (e.g. hospital records)* | No |  | No | Yes* |  | Medical records* | No description | Complete follow - all subjects accounted for* | Average |
| Dettinger 2020 | Somewhat representative of the pregnant population in the community | The comparator cohort is drawn from the same community as the exposed cohort* | PrEP intake confirmed from secure medical records (e.g. hospital records)* | Yes* |  | No | Yes* |  | Medical records* | No description | Subjects lost to follow-up unlikely to introduce bias i.e. <20% lost to follow-up* | Average |
| Heffron 2018 | Somewhat representative of the pregnant population in the community | The comparator cohort is drawn from a different source than the exposed cohort | PrEP intake monitored as part of the study* | Yes* |  | No | Yes* |  | Medical records* | No description | Complete follow - all subjects accounted for* | Average |
| Matthews 2018 | No description | The comparator cohort is drawn from the same community as the exposed cohort* | No description | Yes* |  | No | No |  | No description | No description | Follow-up rate <80% (LTFU >20%) | Poor |
| Each (*) indicates one point towards category score | | | | | | | | | | | | |

## Appendix 4.4: Confounding factors adjusted for in included cohort studies

| **Study** | **Methods to account for potential confounding** | **Risk factor analysis:**  **No statistically significant difference between groups** | **Risk factor analysis:**  **Statistically significant difference between groups** | **Regression analysis:**  **Characteristics adjusted for in multivariable regression** |
| --- | --- | --- | --- | --- |
| Davey 2022 | Risk factor analysis | Maternal age; Education; Relationship status; Gestational age at PrEP offer; STI diagnosis; More than one sex partner in the past 12 months; Substance use in the past 12 months; Partner HIV test result in the past 12 months; HIV self-risk perception | Gravidity; Receiving STI treatment same day as diagnosis; experienced IPV in past 12 months | Not conducted |
| Dettinger 2019 | Risk factor analysis and regression | Age; Marital status; Couple tested for HIV together during ANC; Engaged in sex in exchange of money/favours; diagnosed with or treated for an STI; Forced to have sex; Shared needles while engaging in IVD; used PEP>=2 times | Gestational age at PrEP screening; Partner HIV status; Syphilis test result; Ever had sex without a condom; Experienced IPV | Gestational age at PrEP screening; Partner HIV status |
| Dettinger 2020 | Risk factor analysis and regression | Gestational age at enrolment | Maternal age; Partner HIV status; Lifetime number of sex partners: Syphilis test result; Transactional sex experience; Diagnosed with or treated for an STI; Forced to have sex against your will; IPV; Physical assault history | Maternal age; Partner HIV status; Syphilis test result |
| Heffron 2018 | Risk factor analysis and regression | Prior pregnancy loss; PTB history | Maternal age; Nulliparity; HIV risk score (no p-value given; author reported difference) | Maternal age; History of pregnancy loss |
| Matthews 2018 | None | Not conducted | Not conducted | Not conducted |
| Abbreviations: ANC, antenatal clinic; IPV, intimate partner violence; IVD, intravenous drugs; PEP, post-exposure prophylaxis; PrEP, pre-exposure prophylaxis; PTB, preterm birth; STI, sexually transmitted infection. | | | | |

# APPENDIX 5: Fixed-effect meta-analyses of the association between PrEP exposure during pregnancy and adverse perinatal and maternal outcomes.

a)


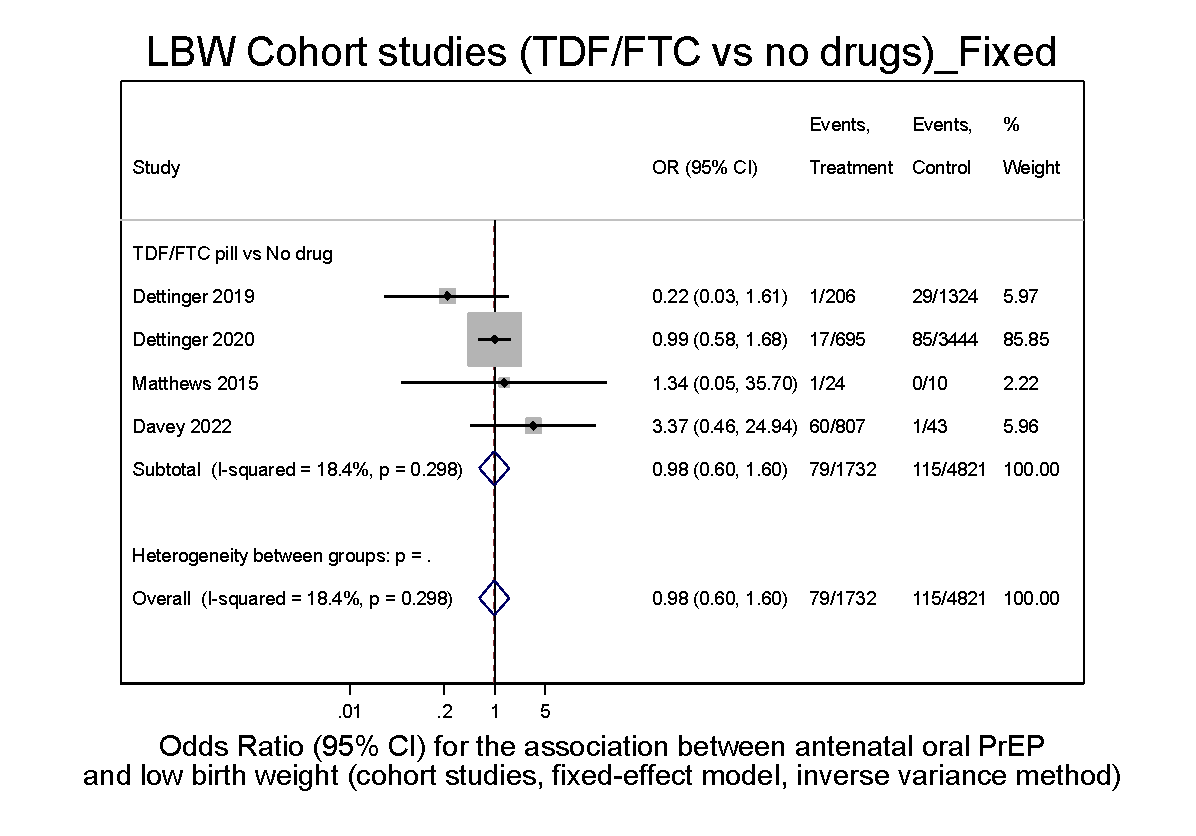


b)


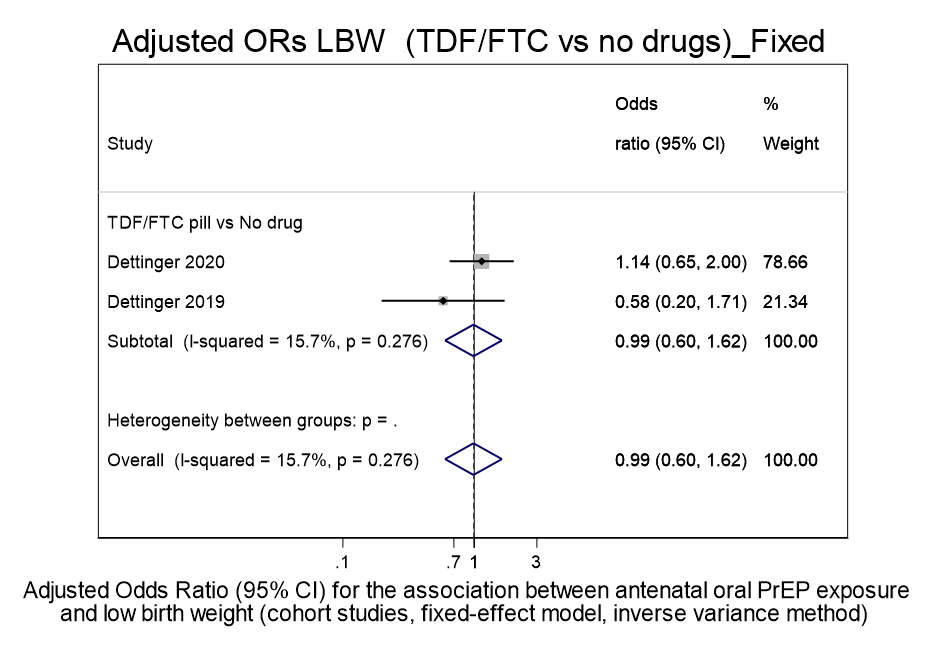


## Appendix 5.1: Cohort studies assessing the association between low birthweight (LBW) and oral PrEP exposure, compared to no drug.

Meta-analyses of a) unadjusted and b) adjusted cohort studies (fixed-effect model, inverse variance method). Grey shaded boxes display the relative contribution (% weight) of each individual study to the meta-analysis. OR, Odds Ratios (95% confidence intervals), number of LBW events and total live births by arm (treatment and control), weighting % are displayed. Abbreviations: TDF, tenofovir disoproxil fumarate; FTC, emtricitabine.

a)


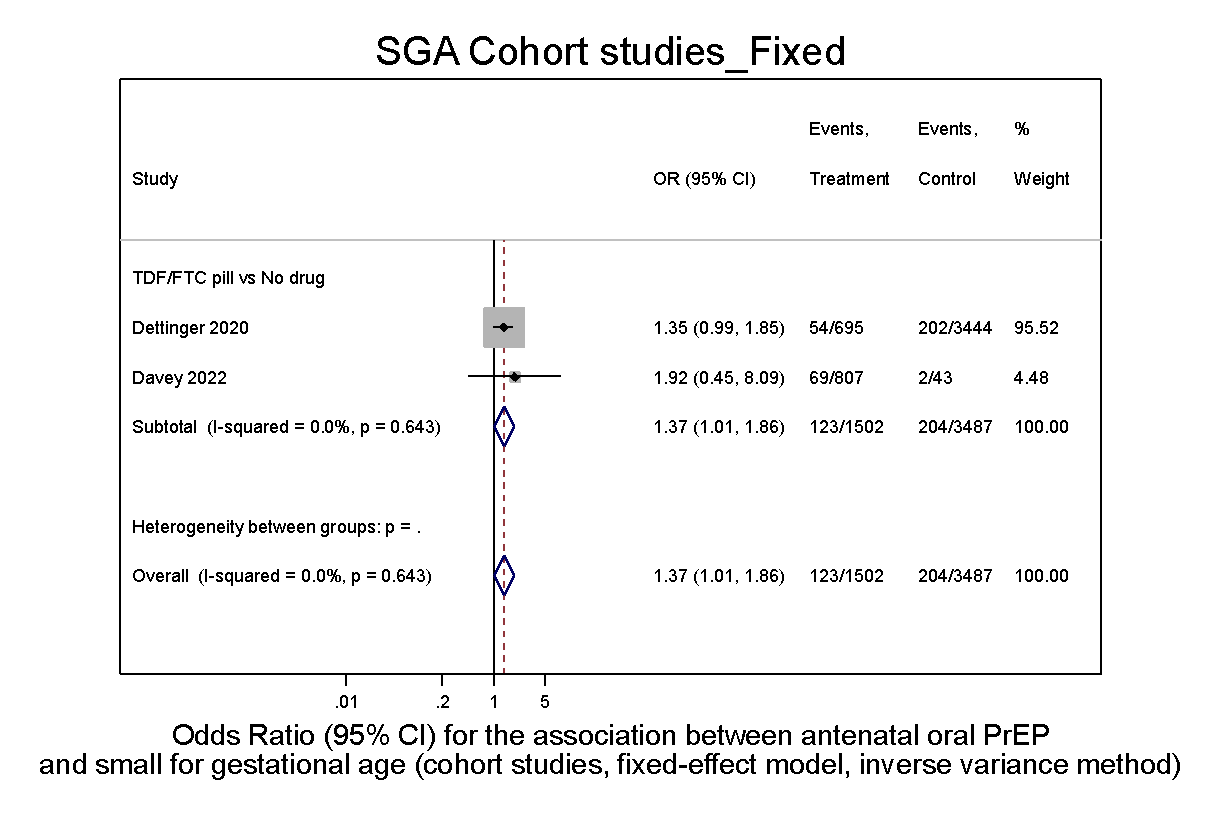


b)
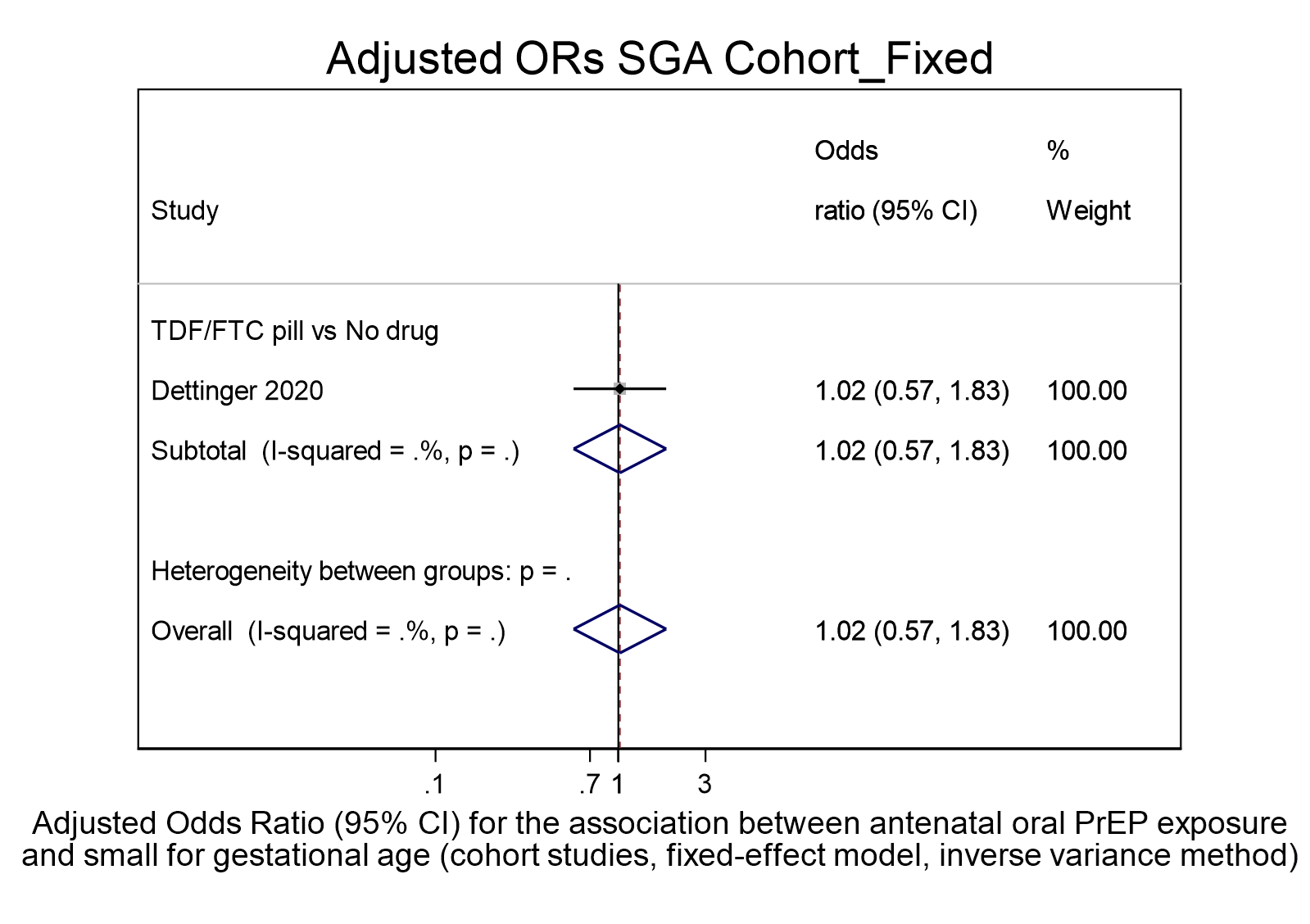


## Appendix 5.2: Cohort studies assessing the association between small for gestational age (SGA) and oral PrEP exposure, compared to no drug.

Meta-analyses of a) unadjusted and b) adjusted cohort studies (fixed-effect model, inverse variance method). Grey shaded boxes display the relative contribution (% weight) of each individual study to the meta-analysis. OR, Odds Ratios (95% confidence intervals), number of SGA events and total live births by arm (treatment and control), weighting % are displayed. Abbreviations: TDF, tenofovir disoproxil; FTC, emtricitabine.


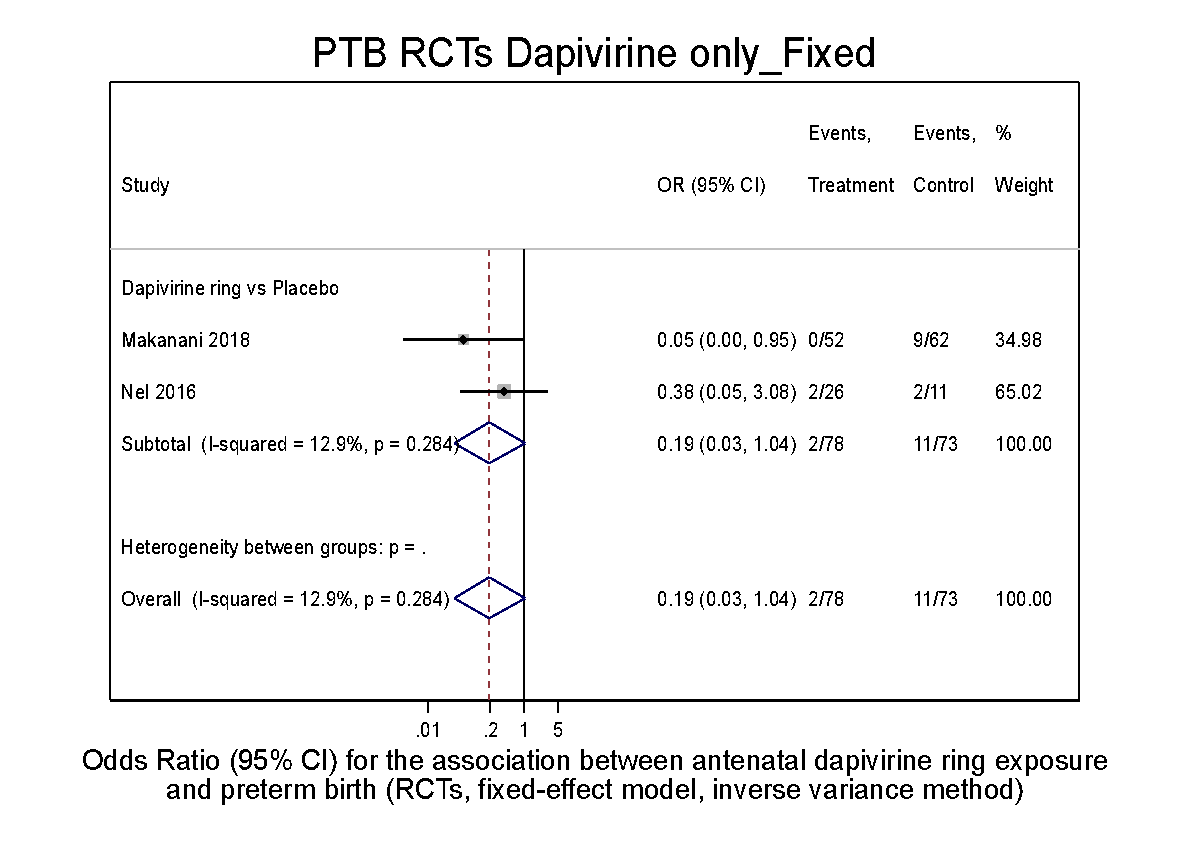


## Appendix 5.3: RCTs assessing the association between preterm birth (PTB) and Dapivirine ring exposure, compared to placebo.

Meta-analysis of RCTs (fixed-effect model, inverse variance method). Grey shaded boxes display the relative contribution (% weight) of each individual study to the meta-analysis. OR, Odds Ratios (95% confidence intervals), number of PTB events and total live births by arm (treatment and control), weighting % are displayed.


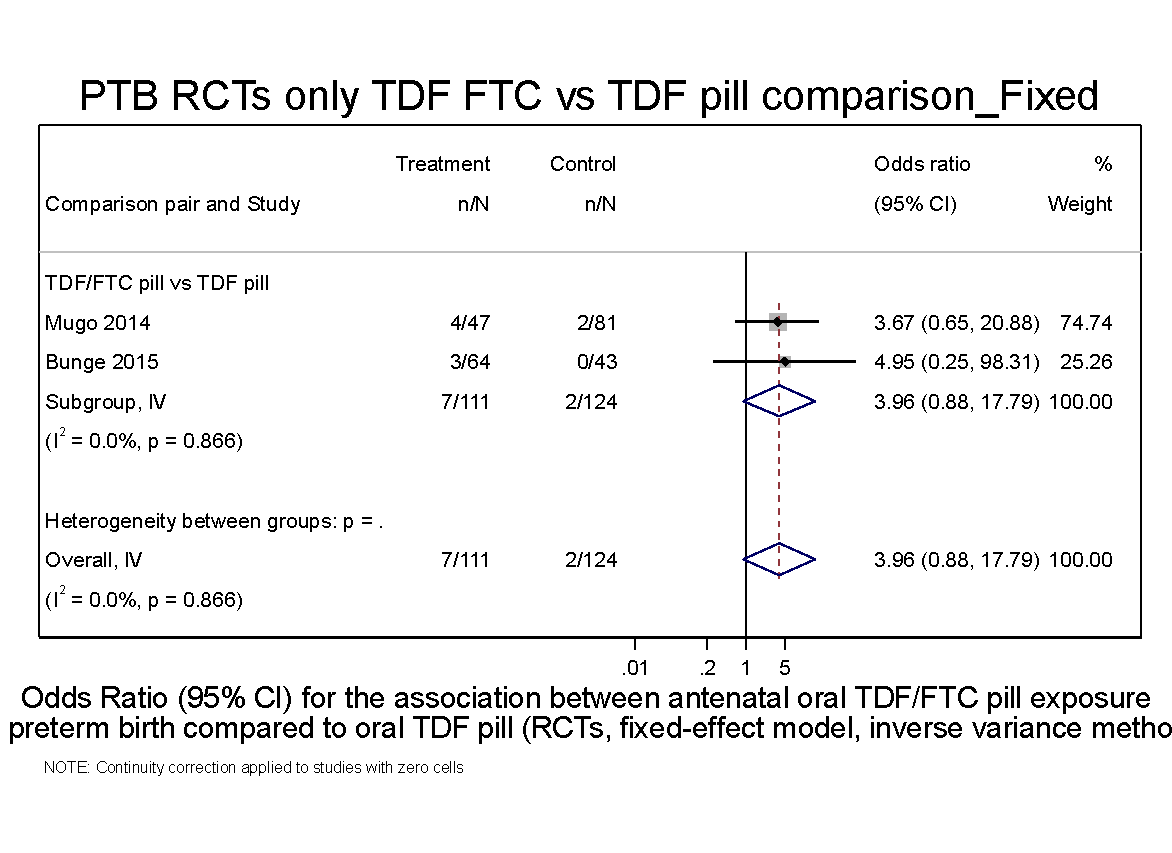


## Appendix 5.4: RCTs assessing preterm birth (PTB) and exposure to TDF/FTC oral pill, compared to TDF oral pill.

Meta-analysis of RCTs (fixed-effect model, inverse variance method). Grey shaded boxes display the relative contribution (% weight) of each individual study to the meta-analysis. OR, Odds Ratios (95% confidence intervals), number of PTB events and total live births by arm (treatment and control), weighting % are displayed.

# Appendix 6: Summary of outcomes from included studies

## Appendix 6.1: Odds ratios.

Perinatal and maternal HIV infection outcomes (Odds Ratios and 95% CI) associated with using HIV pre-exposure prophylaxis during pregnancy.

|  | **OR (95% CI)** | | | | | | |
| --- | --- | --- | --- | --- | --- | --- | --- |
|  | **PTB** | **LBW** | **vLBW** | **preterm LBW** | **SGA** | **Neonatal death** | **Maternal HIV infection** |
| **Randomised controlled trials** | | | | | | | |
| Oral PrEP vs no oral PrEP | 0.73  (0.43-1.26) | 1.41  (0.72, 2.76) | 1.00  (0.14,7.16) |  | 1.63  (0.38, 6.90) | 0.14  (0.01, 2.71) | 3.00  (0.31, 29.02) |
| Dapivirine ring vs placebo | 0.19  (0.03, 1.04) |  |  |  |  | 1.00  (0.03, 30.62) |  |
| TFV gel (1.0%) vs placebo | 1.54  (0.47-5.02) |  |  |  |  |  |  |
| TDF/FTC pill vs TDF pill | 3.96  (0.88-17.79) |  |  |  |  |  |  |
| TDF/FTC pill vs TFV gel (1.0%) | 0.31  (0.08-1.24 |  |  |  |  |  |  |
| TDF pill vs TFV gel (1.0%) | 0.07  (0.00-1.24) |  |  |  |  |  |  |
| TDF/FTC pill vs Dapivirine ring | 1.80  (0.59-5.50)) |  |  |  |  | 2.05  (0.13-33.14) |  |
| **Randomised controlled trials sub-group analysis by oral PrEP type** | | | | | | | |
| TDF/FTC pill versus no drug | 0.92  (0.45,1.92) | 1.41  (0.72, 2.76) | 1.00  (0.14,7.16) |  | 1.63  (0.38, 6.90) | 0.14  (0.01, 2.71) | 3.00  (0.31, 29.02) |
| TDF/FTC pill vs placebo | 0.81  (0.31, 2.08) |  |  |  |  |  |  |
| TDF pill vs placebo | 0.24  (0.06, 1.01) |  |  |  |  |  |  |
| **Cohort studies** | | | | | | | |
| Oral PrEP vs no oral PrEP (unadjusted) | 0.84  (0.69, 1.03) | 0.98  (0.60, 1.60) |  | 1.34  (0.05, 35.70) | 1.37  (1.01, 1.86) | 0.49  (0.03, 9.19) | 0.21  (0.01, 3.81) |
| Oral PrEP vs no oral PrEP (adjusted) | 0.67  (0.52, 0.88) | 0.99  (0.60, 1.62) |  |  | 1.02  (0.57, 1.83) |  |  |
| **Cohort studies sub-group analysis by oral PrEP type (Unadjusted analyses)** | | | | | | | |
| TDF/FTC pill versus no drug | 0.85  (0.69, 1.04) | 0.98  (0.60, 1.60) |  | 1.34  (0.05, 35.70) | 1.37  (1.01, 1.86) | 0.49  (0.03, 9.19) |  |
| TDF/FTC pill vs placebo | 0.22  (0.01, 4.05) |  |  |  |  |  | 0.21  (0.01, 3.81) |
| **Cohort studies sub-group analysis by oral PrEP type (Adjusted analyses)** | | | | | | | |
| TDF/FTC pill versus no drug | 0.68  (0.52, 0.89) | 0.99  (0.60, 1.62) |  |  | 1.02  (0.57, 1.83) |  |  |
| TDF/FTC pill vs placebo | 0.59  (0.15, 2.27) |  |  |  |  |  |  |

## Appendix 6.2: Number of studies.

Number of studies included to evaluate the association between HIV pre-exposure prophylaxis use during pregnancy and perinatal and maternal HIV infection outcomes.

|  | **Number of studies included** | | | | | | |
| --- | --- | --- | --- | --- | --- | --- | --- |
|  | **PTB** | **LBW** | **vLBW** | **preterm LBW** | **SGA** | **Neonatal death** | **Maternal HIV infection** |
| **Randomised controlled trials** | | | | | | | |
| Oral PrEP vs no oral PrEP | 6 | 1 | 1 |  | 1 | 1 | 1 |
| Dapivirine ring vs placebo | 3 |  |  |  |  | 1 |  |
| TFV gel (1.0%) vs placebo | 1 |  |  |  |  |  |  |
| TDF/FTC pill vs TDF pill | 2 |  |  |  |  |  |  |
| TDF/FTC pill vs TFV gel (1.0%) | 1 |  |  |  |  |  |  |
| TDF pill vs TFV gel (1.0%) | 1 |  |  |  |  |  |  |
| TDF/FTC pill vs Dapivirine ring | 1 |  |  |  |  | 1 |  |
| **Randomised controlled trials sub-group analysis by oral PrEP type** | | | | | | | |
| TDF/FTC pill versus no drug | 1 | 1 | 1 |  | 1 | 1 | 1 |
| TDF/FTC pill vs placebo | 3 |  |  |  |  |  |  |
| TDF pill vs placebo | 2 |  |  |  |  |  |  |
| **Cohort studies** | | | | | | | |
| Oral PrEP (unadjusted) | 5 | 4 |  | 1 | 2 | 1 | 1 |
| Oral PrEP (adjusted) | 3 | 2 |  |  | 1 |  |  |
| **Cohort studies sub-group analysis by oral PrEP type (Unadjusted analyses)** | | | | | | | |
| TDF/FTC pill versus no drug | 4 | 4 |  | 1 | 2 | 1 |  |
| TDF/FTC pill vs placebo | 1 |  |  |  |  |  | 1 |
| **Cohort studies sub-group analysis by oral PrEP type (Adjusted analyses)** | | | | | | | |
| TDF/FTC pill versus no drug | 2 | 2 |  |  | 1 |  |  |
| TDF/FTC pill vs placebo | 1 |  |  |  |  |  |  |

## Appendix 6.3: Number of women analysed.

Total number of women analysed as part of the meta-analysis to evaluate the association between HIV pre-exposure prophylaxis use during pregnancy and perinatal and maternal HIV infection outcomes.

|  | **Number of women analyzed** | | | | | | |
| --- | --- | --- | --- | --- | --- | --- | --- |
|  | **PTB** | **LBW** | **vLBW** | **preterm LBW** | **SGA** | **Neonatal death** | **Maternal HIV infection** |
| **Randomised controlled trials** | | | | | | | |
| Oral PrEP vs no oral PrEP | 1,047 | 474 | 474 |  | 453 | 481 | 540 |
| Dapivirine ring vs placebo | 151 |  |  |  |  | 14 |  |
| TFV gel (1.0%) vs placebo | 113 |  |  |  |  |  |  |
| TDF/FTC pill vs TDF pill | 235 |  |  |  |  |  |  |
| TDF/FTC pill vs TFV gel (1.0%) | 123 |  |  |  |  |  |  |
| TDF pill vs TFV gel (1.0%) | 102 |  |  |  |  |  |  |
| TDF/FTC pill vs Dapivirine ring | 301 |  |  |  |  | 301 |  |
| **Randomised controlled trials sub-group analysis by oral PrEP type** | | | | | | | |
| TDF/FTC pill versus no drug | 478 | 474 | 474 |  | 453 | 481 | 540 |
| TDF/FTC pill vs placebo | 314 |  |  |  |  |  |  |
| TDF pill vs placebo | 255 |  |  |  |  |  |  |
| **Cohort studies** | | | | | | | |
| Oral PrEP (unadjusted) | 6,643 | 6,553 |  | 34 | 4,989 | 850 | 118 |
| Oral PrEP (adjusted) | 5,759 | 5,669 |  |  | 4,139 |  |  |
| **Cohort studies sub-group analysis by oral PrEP type (Unadjusted analyses)** | | | | | | | |
| TDF/FTC pill versus no drug | 6,553 | 6,553 |  | 34 | 4,989 | 850 |  |
| TDF/FTC pill vs placebo | 90 |  |  |  |  |  | 118 |
| **Cohort studies sub-group analysis by oral PrEP type (Adjusted analyses)** | | | | | | | |
| TDF/FTC pill versus no drug | 5,669 | 5,669 |  |  | 4,139 |  |  |
| TDF/FTC pill vs placebo | 90 |  |  |  |  |  |  |

#

# APPENDIX 7: Funnel plots to assess for small study effects.

Funnel plots to assess for small study effects in meta-analyses assessing the association between perinatal outcomes and exposure to HIV PrEP during pregnancy, compared to no PrEP exposure.


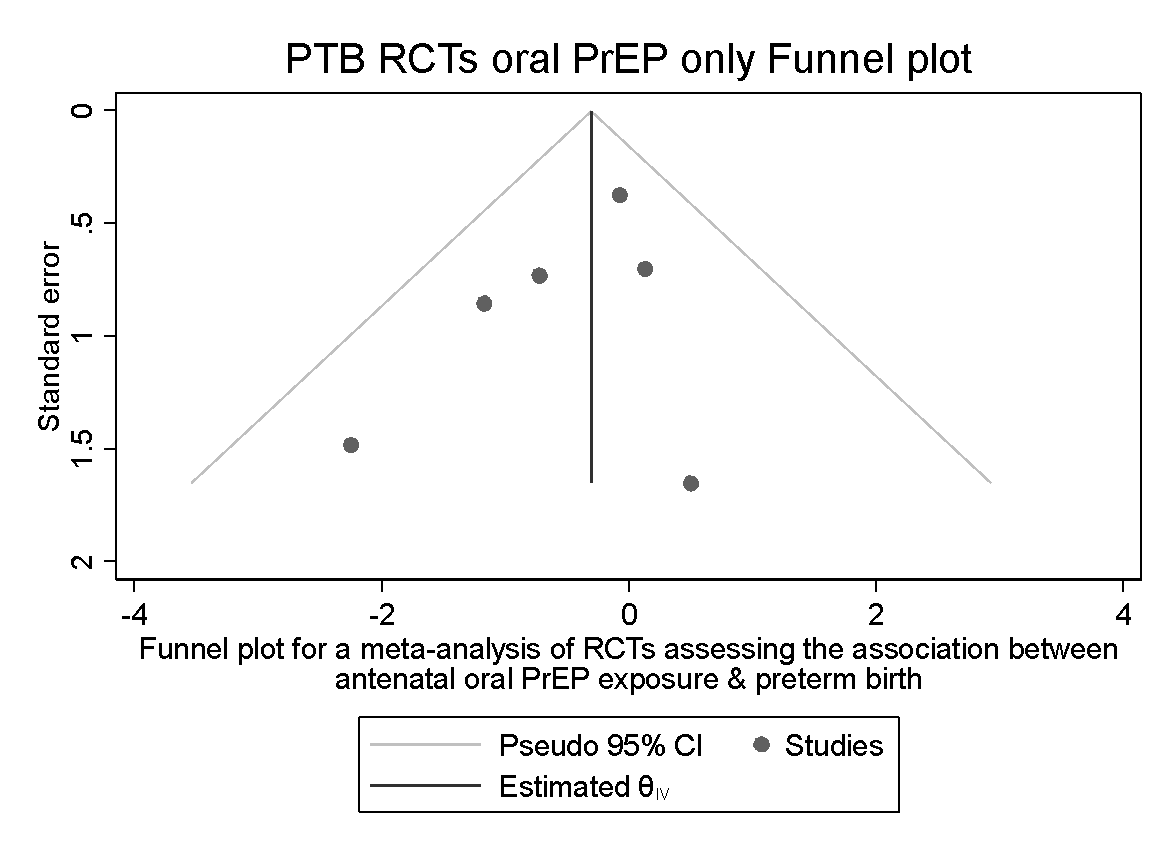


## Appendix 7.1: RCTs assessing the association between preterm birth (PTB) and oral PrEP exposure during pregnancy vs no oral PrEP


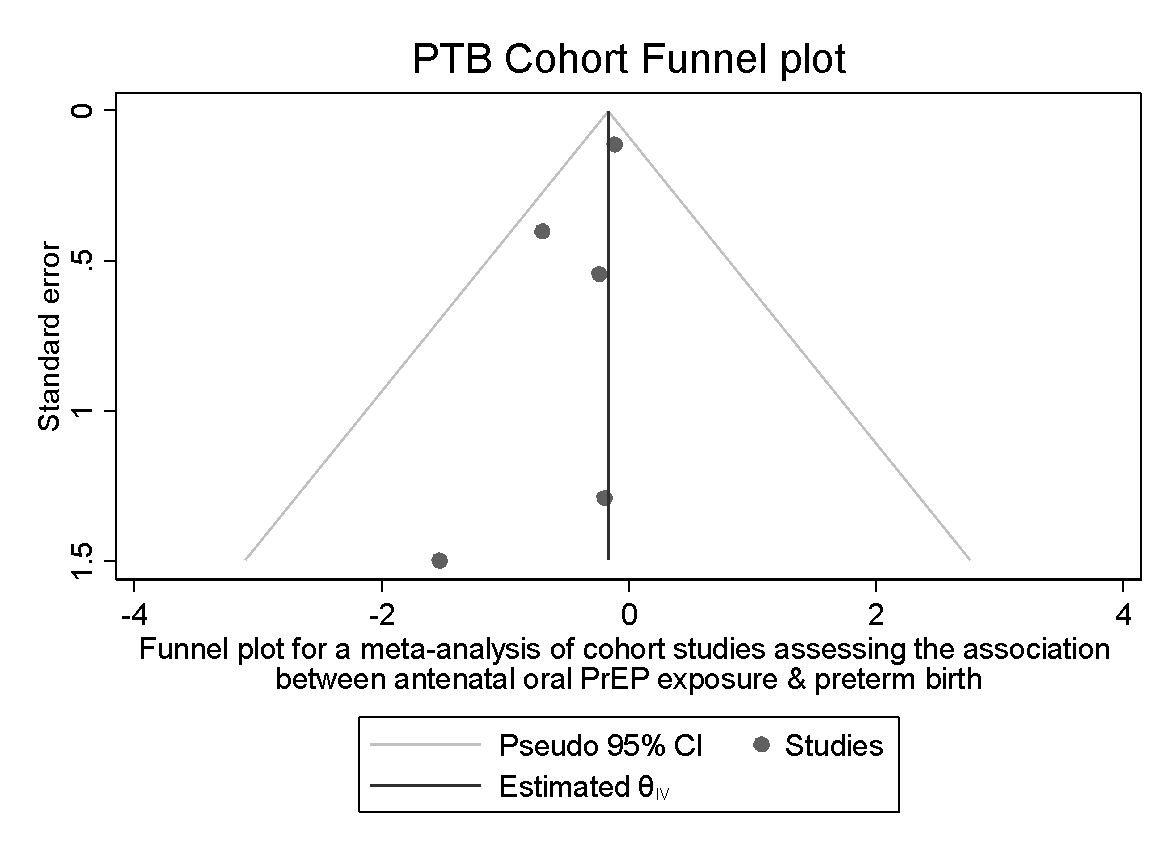


## Appendix 7.2: Cohort studies assessing the association between preterm birth (PTB) and oral PrEP exposure.


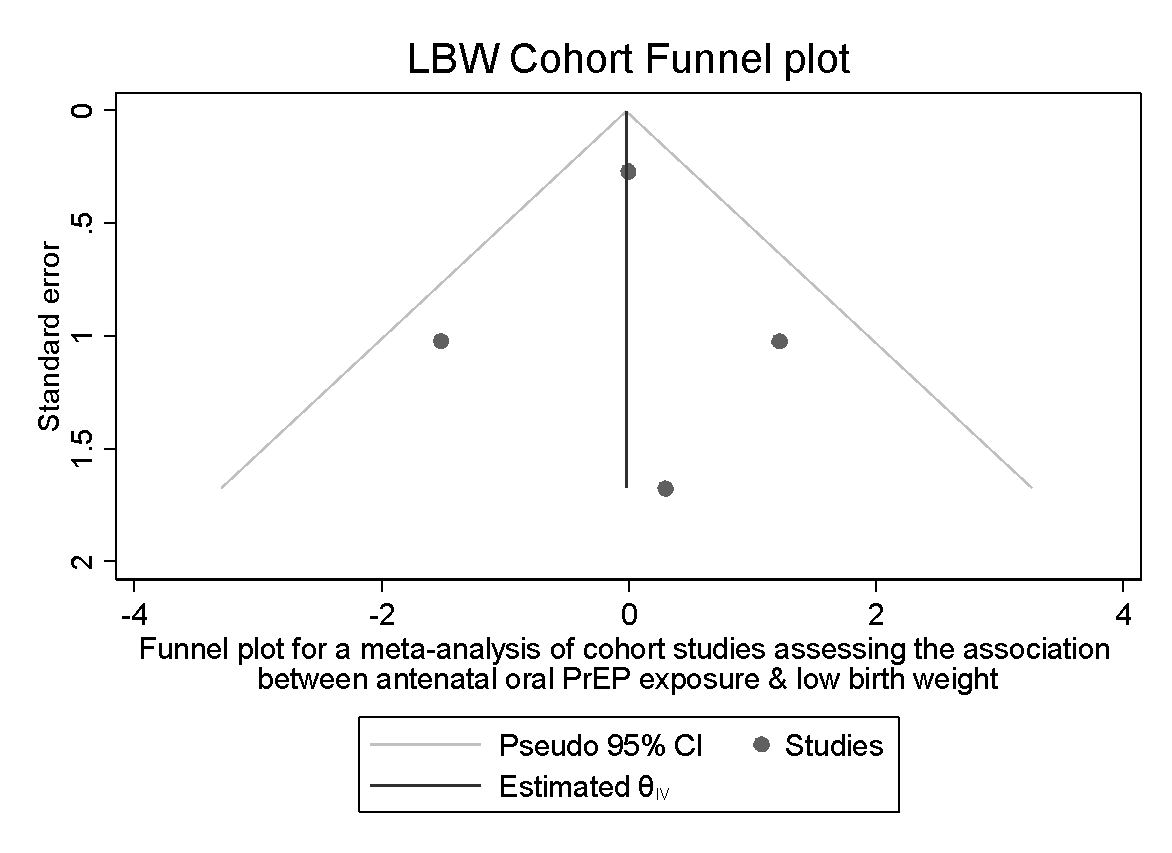


## Appendix 7.3: Cohort studies assessing the association between low birth weight (LBW) and oral PrEP exposure, compared to no PrEP exposure.


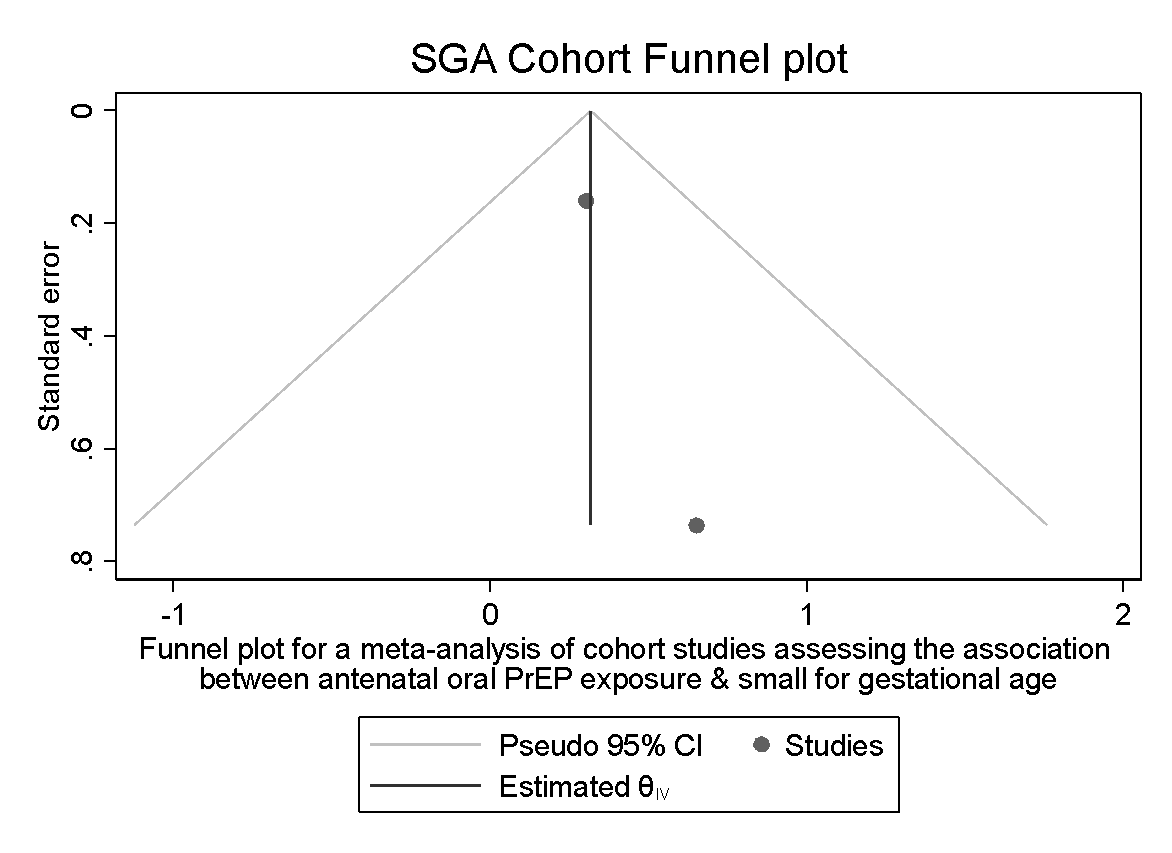


## Appendix 7.4: Cohort studies assessing the association between small for gestational age and oral PrEP exposure during pregnancy, compared to no PrEP exposure.


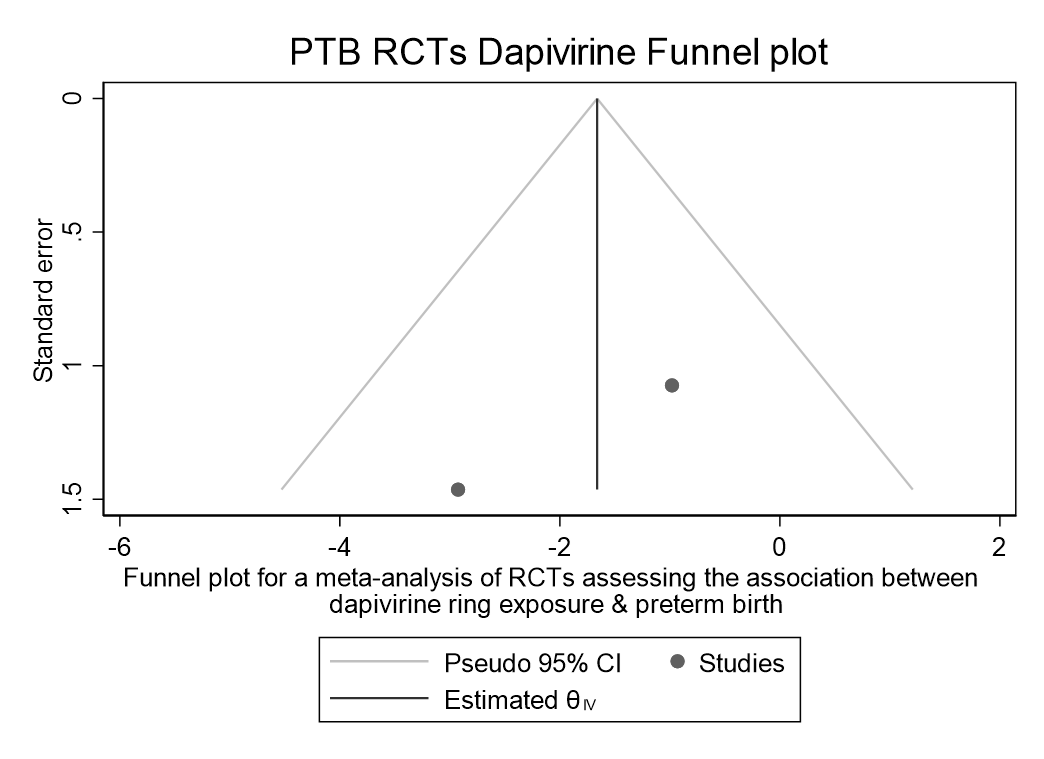


## Appendix 7.5: RCTs assessing the association between preterm birth and dapivirine ring exposure, compared to no dapivirine ring


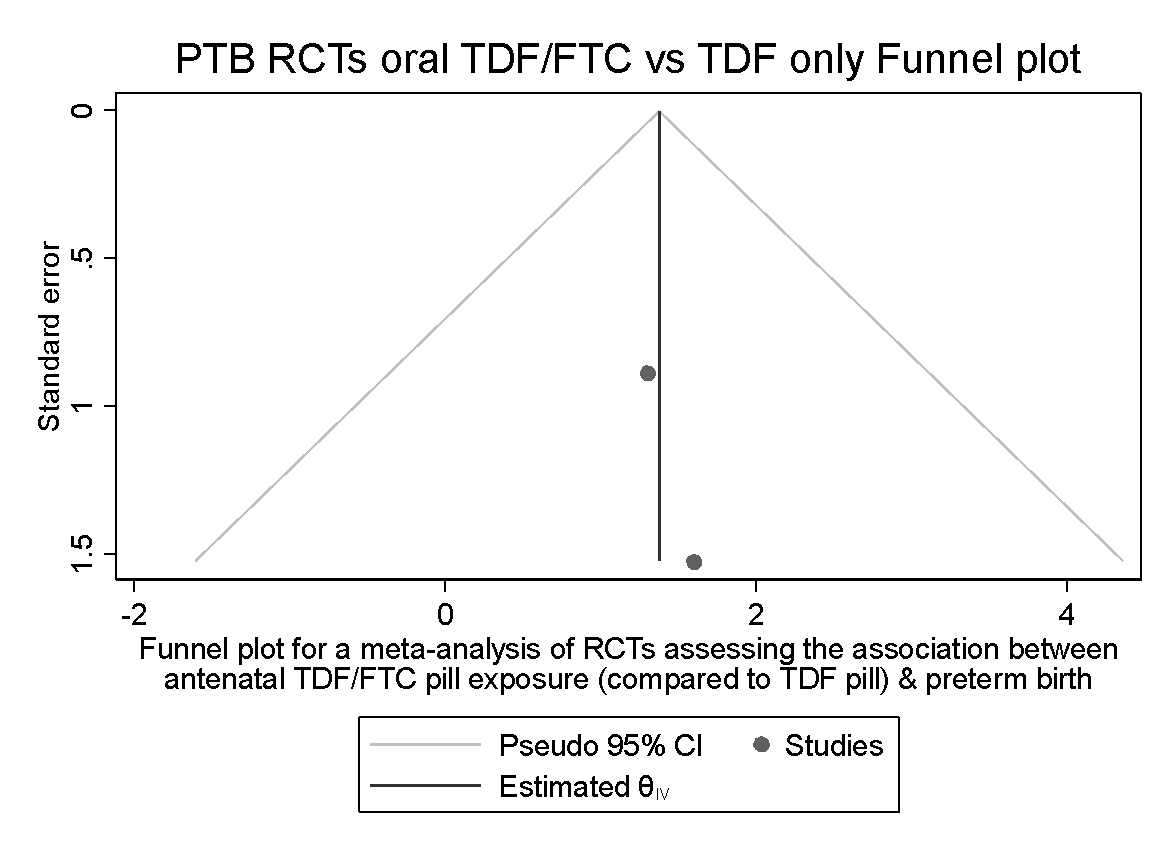


## Appendix 7.6: RCTs assessing the association between preterm birth and TDF/FTC oral pill, compared to TDF oral pill.

#

# APPENDIX 8: Random effects meta-analyses of the association between PrEP exposure during pregnancy and adverse perinatal and maternal outcomes.


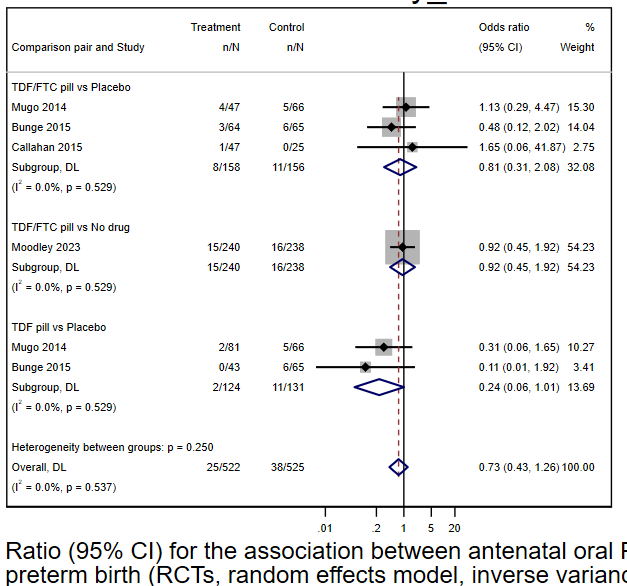


## Appendix 8.1: RCTs assessing the association between preterm birth (PTB) and oral PrEP exposure

Meta-analysis of RCTs (random effects model, inverse variance method). Grey shaded boxes display the relative contribution (% weight) of each individual study to the meta-analysis. Number of PTB events and total live births by arm (treatment and control), Odds Ratios (95% confidence intervals), weighting % are displayed.

a)


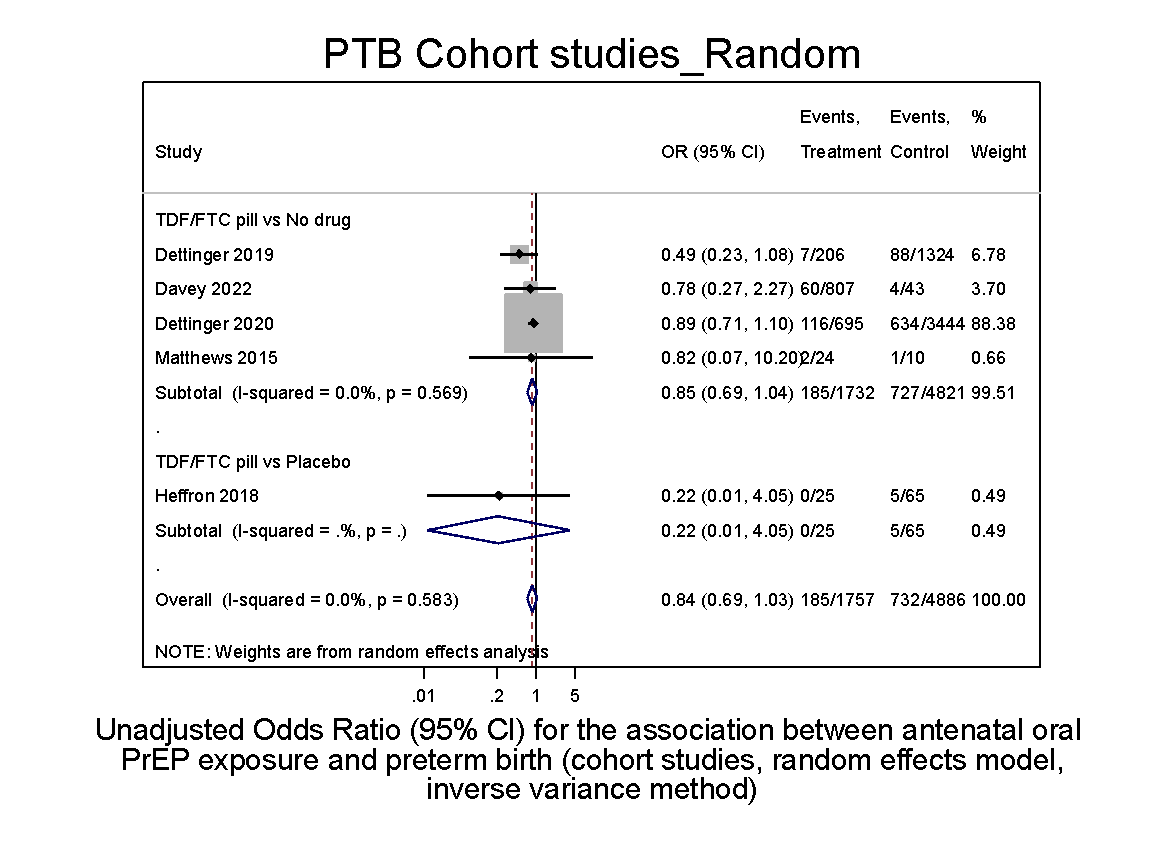


b)
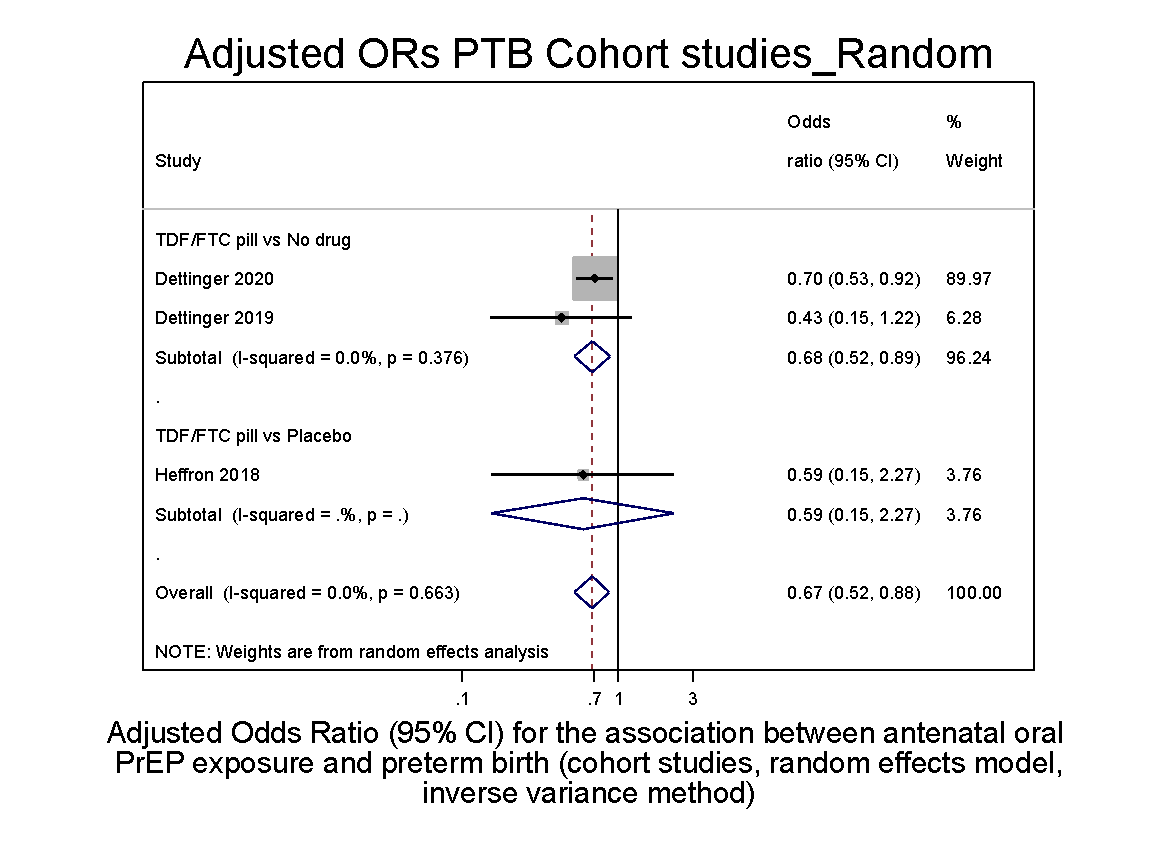


## Appendix 8.2: Cohort studies assessing the association between preterm birth (PTB) and oral PrEP exposure

Meta-analysis of a) unadjusted and b) adjusted cohort studies (random effects model, inverse variance method). Grey shaded boxes display the relative contribution (% weight) of each individual study to the meta-analysis. Odds Ratios (95% confidence intervals), weighting % are displayed.

a)


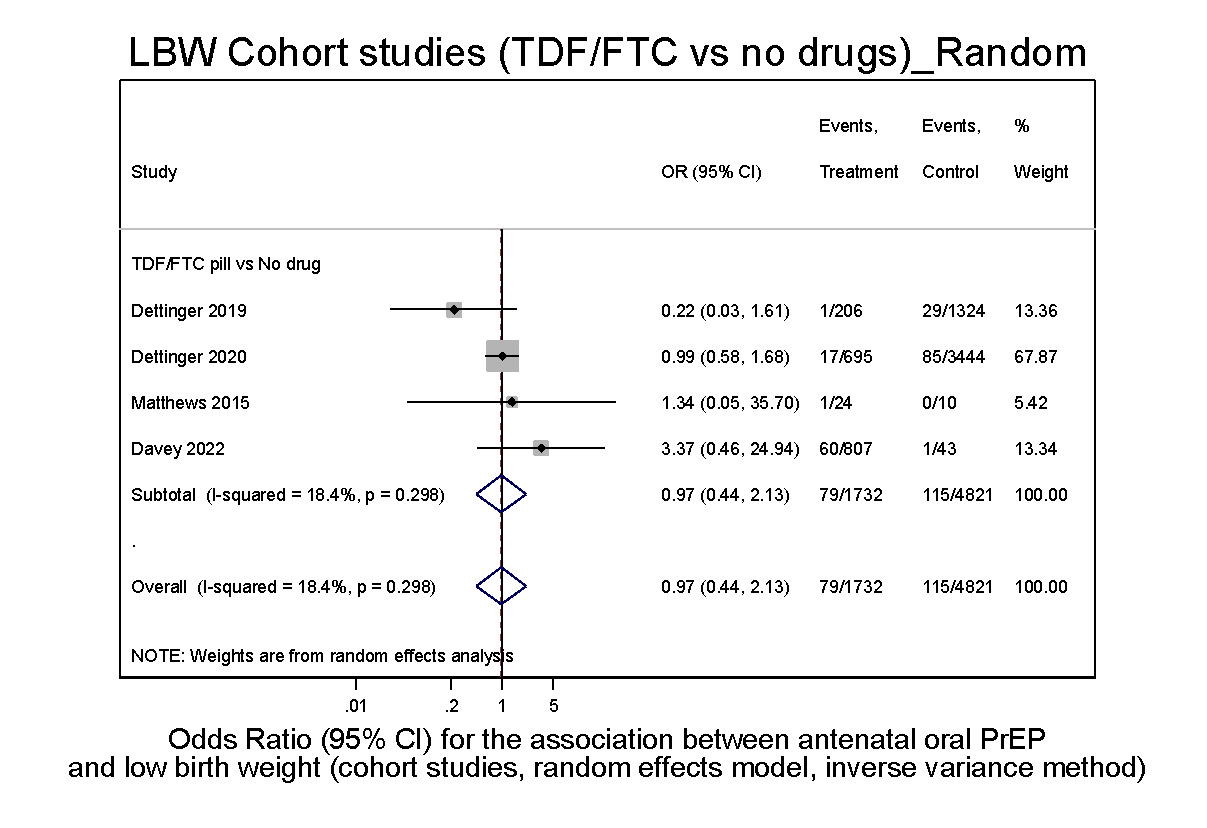


b)


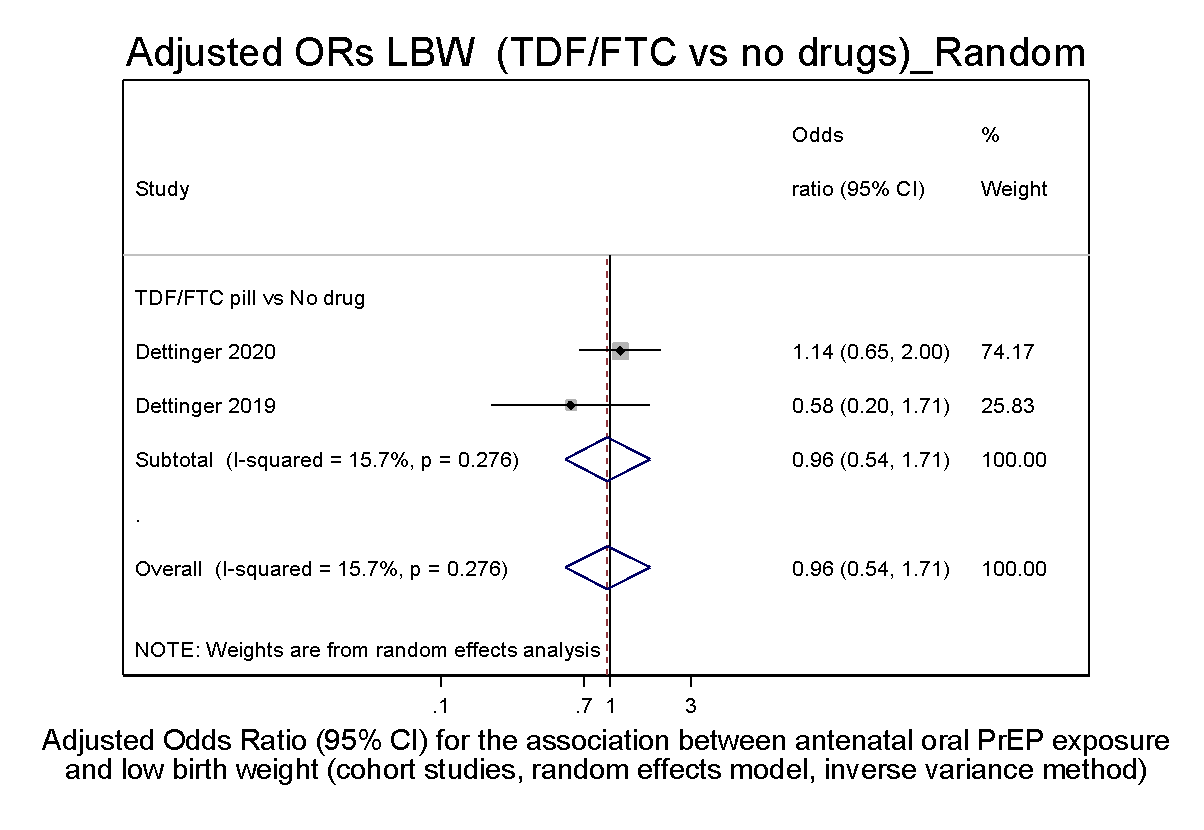


## Appendix 8.3: Cohort studies assessing the association between low birthweight (LBW) and oral PrEP exposure

Meta-analysis of a) unadjusted and b) adjusted cohort studies (random effects model, inverse variance method). Grey shaded boxes display the relative contribution (% weight) of each individual study to the meta-analysis. Odds Ratios (95% confidence intervals), weighting % are displayed.

a)


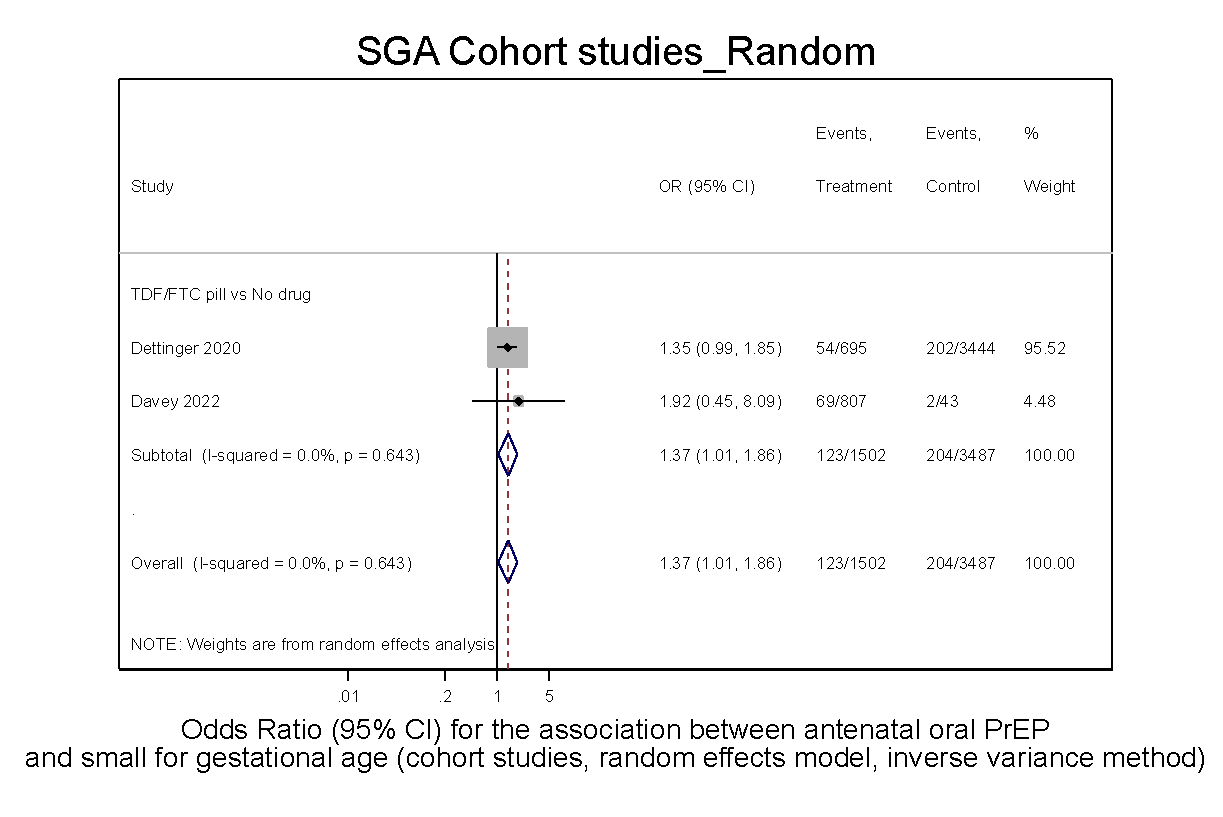


b)


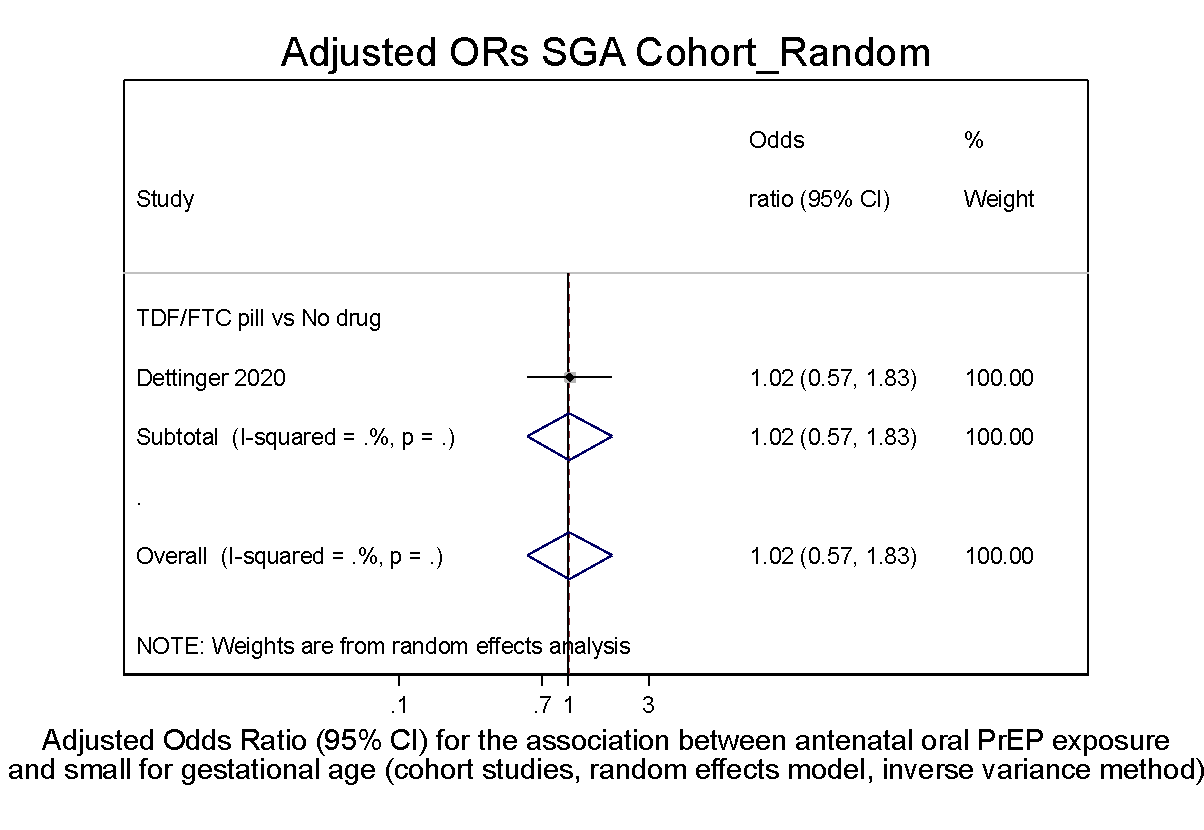


## Appendix 8.4: Cohort studies assessing the association between small for gestational age (SGA) and oral PrEP exposure

Meta-analysis of a) unadjusted and b) adjusted cohort studies (random effects model, inverse variance method). Grey shaded boxes display the relative contribution (% weight) of each individual study to the meta-analysis. Odds Ratios (95% confidence intervals), weighting % are displayed.


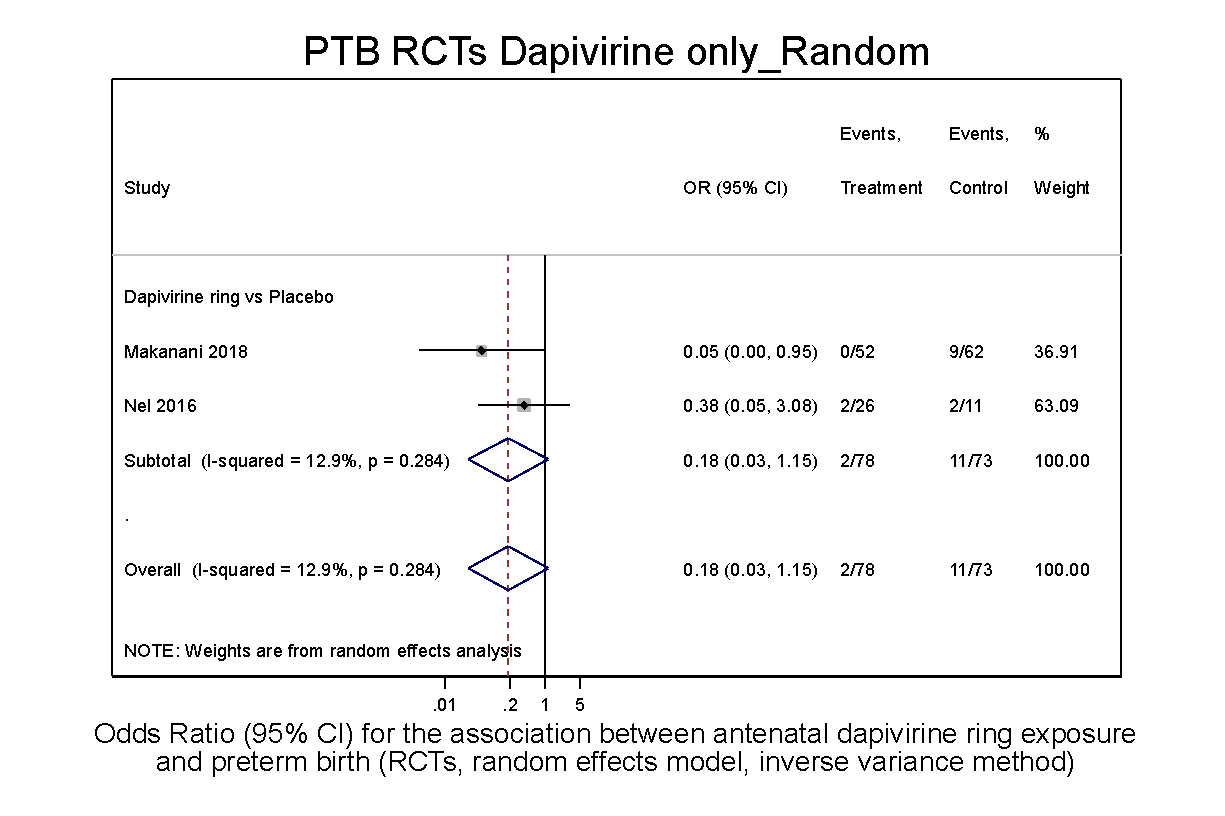


## Appendix 8.5: RCTs assessing the association between preterm birth (PTB) and dapivirine ring exposure

Meta-analysis of RCTs (random effects model, inverse variance method). Grey shaded boxes display the relative contribution (% weight) of each individual study to the meta-analysis. OR, Odds Ratios (95% confidence intervals), number of PTB events and total live births by arm (treatment and control), weighting % are displayed.
